# Health and health system impacts of China’s comprehensive primary healthcare reforms: a systematic review

**DOI:** 10.1093/heapol/czad058

**Published:** 2023-07-28

**Authors:** Chang Cai, Shangzhi Xiong, Christopher Millett, Jin Xu, Maoyi Tian, Thomas Hone

**Affiliations:** Public Health Policy Evaluation Unit, School of Public Health, Imperial College London, Reynolds Building, St Dunstan's Road, London W6 8RP, UK; The George Institute for Global Health, Faulty of Medicine and Health, University of New South Wales, Level 5, 1 King Street Newtown, Sydney 2042, Australia; Global Health Research Centre, Duke Kunshan University, Academic Building 3038, No. 8 Duke Avenue, Kunshan, Jiangsu 215316, China; Public Health Policy Evaluation Unit, School of Public Health, Imperial College London, Reynolds Building, St Dunstan's Road, London W6 8RP, UK; Public Health Research Centre and Comprehensive Health Research Centre, NOVA National School of Public Health, NOVA University Lisbon, Avenida Padre Cruz, Lisbon 1600-560, Portugal; China Center for Health Development Studies, Peking University Health Science Center, 38 Xueyuan Road, Haidian District, Beijing 100191, China; The George Institute for Global Health, Faulty of Medicine and Health, University of New South Wales, Level 5, 1 King Street Newtown, Sydney 2042, Australia; School of Public Health, Harbin Medical University, No. 157 Baojian Road, Nangang District, Harbin 150081, China; Public Health Policy Evaluation Unit, School of Public Health, Imperial College London, Reynolds Building, St Dunstan's Road, London W6 8RP, UK

**Keywords:** Primary health care, China, health reforms, policy evaluations

## Abstract

China’s comprehensive primary healthcare (PHC) reforms since 2009 aimed to deliver accessible, efficient, equitable and high-quality healthcare services. However, knowledge on the system-wide effectiveness of these reforms is limited. This systematic review synthesizes evidence on the reforms’ health and health system impacts. In 13 August 2022, international databases and three Chinese databases were searched for randomized controlled trials, quasi-experimental studies and controlled before-after studies. Included studies assessed large-scale PHC policies since 2009; had a temporal comparator and a control group and assessed impacts on expenditures, utilization, care quality and health outcomes. Study quality was assessed using Risk of Bias In Non-randomized Studies of Interventions, and results were synthesized narratively. From 49 174 identified records, 42 studies were included—all with quasi-experimental designs, except for one randomized control trial. Nine studies were assessed as at low risk of bias. Only five low- to moderate-quality studies assessed the comprehensive reforms as a whole and found associated increases in health service utilization, whilst the other 37 studies examined single-component policies. The National Essential Medicine Policy (*N* = 15) and financing reforms (*N* = 11) were the most studied policies, whilst policies on primary care provision (i.e. family physician policy and the National Essential Public Health Services) were poorly evaluated. The PHC reforms were associated with increased primary care utilization (*N* = 17) and improved health outcomes in people with non-communicable diseases (*N* = 8). Evidence on healthcare costs was unclear, and impacts on patients’ financial burden and care quality were understudied. Some studies showed disadvantaged regions and groups that accrued greater benefits (*N* = 8). China’s comprehensive PHC reforms have made some progress in achieving their policy objectives including increasing primary care utilization, improving some health outcomes and reducing health inequalities. However, China’s health system remains largely hospital-centric and further PHC strengthening is needed to advance universal health coverage.

Key messages

- Existing evaluations have mostly focused on single-component primary healthcare (PHC) interventions, and evidence from Asia is lacking. Comprehensive system-wide PHC reforms are limited to few low- and middle-income countries (LMICs), and there is a dearth of evaluations on their synergetic impacts. There is no synthesized understanding of the health system and health impacts of China’s comprehensive PHC reforms starting in 2009.
- China’s comprehensive PHC reforms since 2009 have produced modest impacts including increased primary care utilization, improved the health outcomes of people with non-communicable diseases (NCDs) and disproportionally benefitted vulnerable, high-risk populations and less-resourced regions. The reform impacts on quality of primary care were underexamined, with only a few studies assessing impacts on antibiotic use, satisfaction and perception of care quality among service users. Findings on health service costs were mixed. Substantial evidence gaps remain on the reform impacts on financial protection, general population health and equity.
- PHC-oriented reforms can increase primary care utilization, reduce inequality and improve the health of people with NCDs and should be a priority for LMICs to advance universal health coverage (UHC). Other health and health system impacts remain poorly studied.
- PHC research in LMICs would benefit from robust studies, adequate data on care quality and strengthened knowledge base of holistic PHC reforms to inform policy-making for UHC.

## Introduction

Primary health care (PHC) is the most effective and efficient approach to building strong health systems and achieving universal health coverage (UHC) and the health-related Sustainable Development Goals (Starfield *et al.*, 2005; Hone *et al.*, 2018; World Health Organization, 2018; Kruk and Pate, 2020). However, PHC strengthening in low- and middle-income countries (LMICs) has been challenging due to limited resources, fragmented health systems and poor-quality care (Kruk *et al.*, 2018). These deficits straddle multiple building blocks of health systems and require system-wide reforms (Kruk *et al.*, 2018; Lancet Global Health, 2018). Although policymakers in LMICs are aware of this imperative (Thapa *et al.*, 2019; Kruk and Pate, 2020), the introduction of comprehensive PHC reforms is scarce and there is a dearth of robust evaluations and synthesized evidence (Rule *et al.*, 2014; Lancet Global Health, 2018; Bitton *et al.*, 2019).

China had a hospital-centric health system where public hospitals were the main health service providers and were allocated with a disproportionate share of resources, whilst the PHC sector was under-developed. In 2008, hospitals in China accounted for 53.1% of total health expenditures, employed 54.3% of doctors and delivered 53.4% of outpatient care (Ministry of Health, 2009a). In an attempt to change the hospital-dominated system, China initiated wide-reaching health reforms in 2009 aiming to shift to a PHC-oriented health system and deliver accessible, efficient, equitable and quality primary care services to achieve UHC (The State Council of the People's Republic of China, 2009b; Meng and Tang, 2013; Liu *et al.*, 2017). From the perspective of PHC strengthening, this on-going health reform can be divided into two phases: the first phase (between 2009 and 2014) focused on establishing a PHC-based health system and increasing access to primary care services. This included insurance expansion, investment in PHC infrastructure, education and training for medical students and primary care physicians, as well as the introduction of the National Essential Medicine Policy (NEMP) and the National Essential Public Health Services (NEPHS) (Yip *et al.*, 2019). However, low utilization and poor-quality primary care services persisted with little alleviation of the overused hospital system (Meng *et al.*, 2019; Yip *et al.*, 2019). To address these issues, the second phase of the reform starting in 2015 focused on restructuring health service delivery, including establishing a hierarchical, integrated delivery system with partial gatekeeping and the introduction of a primary care model based on family physicians (Table 1) (Fang *et al.*, 2019; Ma *et al.*, 2019; Meng *et al.*, 2019; Yuan *et al.*, 2019). It is worth noting that this PHC reform is part of wider reforms, including those in the public hospital system (Liu *et al.*, 2011; Yip *et al.*, 2012).

**Table 1.** A summary for major China’s PHC policies since 2009

Policy	Content
The first phase of the PHC reform (2009–2014)	
Infrastructure strengthening (The State Council of the People's Republic of China, 2009a; The State Council of the People's Republic of China, 2009b; The State Council of the People's Republic of China, 2011) (March 2009)	Provided new public funds to build new PHC facilities and refurbish and re-equip existing ones; Introduced new governance models for PHC facilities, including government-regulated PHC facilities, whose funding and revenues are directly managed by local governments, and hospital-affiliated PHC facilities, whose funding and revenues are directly managed by public hospitals; Established a GP system with standardized training programmes for certificated/licenced GPs; Provided subsidies and free training for medical students who are trained as GPs.
Performance reporting (Ministry of Health, 2010; The State Council of the People's Republic of China, 2009a) (June 2010)	Introduced a list of performance indicators for PHC facilities, including antibiotic use, injection prescribing rates and average expenditures for patients; Required PHC facilities to record performance indicators in monthly reports and make monthly reports openly accessible to the public.
Financing reforms (Ministry of Health *et al.*, 2009c; Ministry of Human Resources and Social Security *et al.*, 2009) (July 2009)	Health insurance reforms Increased per-capita subsidies for enrollees of basic health insurance to raise enrolment rates (from 120 Chinese Yuan in 2010 to 610 Chinese Yuan in 2022); Funds for basic health insurance were pooled and managed at city level; Expanded coverage of benefit packages in basic health insurance from hospitalization to primary care services; Reduced co-payment (to about 50%) for costs of outpatient care and medication for people with NCDs (with a cap).
	Provider payment reforms Introduced an evaluation scheme for the performance of PHC facilities, which affects the overall salary of the personnel in each facility. Combined or replaced fee-for-service with P4P or capitation for the delivery of primary care services.
The NEMP (Ministry of Health, 2009b; Ministry of Health *et al.*, 2013) (August 2009)	Created a National Essential Medicine List available at all PHC facilities; Increased the reimbursement rate for all essential medicines; Mandated unified medicine procurement and distribution at the provincial level and engaged local governments in price negotiation, drug bidding, procurement and quality assurance; Removed the mark-up of essential medicines as a revenue source.
The NEPHS (Ministry of Finance, National Population and Family Planning Commission, 2009)(July 2009)	Provided the population with a free-of-charge, defined package of essential health services, including vaccination, health examinations, screening, health management, follow-up visits, prescription and health education (with a priority for people over 65-year-old, with NCDs, 0–6-years old children and pregnant women); Provided extra subsidies to PHC facilities for the delivery of the package based on per-capita allocation; Introduced individual electronic health records for all citizens at all PHC facilities.
**The second phase of the PHC reform (2015–now)**	
Referral system Restructuring (The State Council of the People’s Republic of China, 2015) (September 2015)	Introduced a three-tier health system, including PHC facilities, secondary hospitals and tertiary hospitals, and defined the responsibilities for each level of health facilities; Provided higher reimbursement rates for visits to PHC facilities and referrals from PHC facilities and reduced reimbursement rates or no reimbursement for direct visits to hospitals; Introduced a new gatekeeping policy that requires referral letters from PHC facilities for hospital visits. This policy was not mandatory and implemented according to the local context.
An integrated delivery system (The State Council of the People’s Republic of China, 2017) (April 2017)	Established a standardized, dual referral system between PHC facilities and hospitals to integrate preventive-curative services; Built provider networks to share health resources (e.g. equipment and workforce) and health technology across facilities at different levels; Established an online platform to share electronic health records across facilities, as well as other health information.
Family physician care model (The State Council of the People’s Republic of China, 2016) (May 2016)	Introduced a new care model that featured family physician teams led by GPs or village doctors from PHC facilities, delivering primary care services at the community level; Provided subsidies to family physician teams for services delivered based on capitation;
	Defined a list of basic health services delivered by family physician teams, including health education and examinations, prescription consulting, home visits and personalized care based on patients’ needs; Introduced benefits to patients who are referred by family physicians, including priority to referrals and reservations and higher reimbursement rates for hospital visits.
PHC workforce training (National Health and Family Planning Commission and National Administration of Traditional Chinese Medicine, 2015; The State Council of the People’s Republic of China, 2015) (November 2015)	Provided subsidized job training programmes for workforce at PHC facilities; The provided training excluded medical certification/licences and focused on medical treatment skills, nursing skills, using health information technology, leadership and teamwork.

Abbreviation: GP, general practitioner.

Whilst China’s PHC reforms have been presented and debated extensively, there is no systematic synthesis of their health system and health outcome impacts (Liu *et al.*, 2011; 2017; Yip *et al.*, 2019). This systematic review aims to examine the impacts of China’s PHC reforms since 2009 on the health system and health outcomes based on evaluations, which use robust study designs, including randomized controlled trials, quasi-experimental studies and controlled before-after studies.

## Methods

This systematic review follows a registered protocol (the International Prospective Register of Systematic Reviews registration number CRD42021239991).

### Search strategy

Thirteen international databases and three Chinese databases were searched in August 2022, including MEDLINE, Embase, ScienceDirect, Scopus, Web of Science, the Cumulative Index to Nursing & Allied Health Literature, Cochrane, Ecolit, Jstor, the Healthcare Management Information Consortium, World Bank Library, World Health Organization Library & information networks for knowledge database, Opengrey, the China National Knowledge Infrastructure, Wanfang and cqvip.

The terms ‘primary health care’, ‘family physicians’, ‘ambulatory care’ and their synonyms were searched in titles or abstracts, along with relevant MeSH terms. Some reform-specific terms were also used, such as ‘zero mark-up’ and ‘the National Essential Medicine Policy’. These terms were linked by OR Boolean operators, and the search was further restricted using AND Boolean operators and the words ‘reform’, ‘China’ and their synonyms No language restriction was applied in the search. For studies in Mandarin, similar terms were searched along with specific policy titles, such as ‘Yilianti’ (part of the integrated delivery system, also known as Medical Alliance in Chinese), ‘Fenjizhenliao’ (partial gatekeeping, also known as the Hierarchical Medical System in Chinese) and ‘Xinnonghe’ (abbreviation of New Rural Cooperative Medical Insurance in Chinese). More details on the search strategy can be found in Supplementary Appendix 1. Grey literature was searched in Opengrey. When full-text articles were not available, corresponding authors of the identified studies were contacted for publication information. References of included articles were also screened for additional studies.

### Study selection

Studies were eligible for inclusion if they (1) studied Chinese citizens; (2) evaluated PHC reforms from 2009 described in Table 1 and were implemented at the equivalent of city/county level or above; (3) included both a temporal comparator and a control group with no exposure to the reforms or exposed to alternative interventions [e.g. capitation and pay-for-performance (P4P) for provider payment reforms]; (4) examined outcomes that were PHC-related, including any relevant measures of system and individual health costs, health service utilization, quality of primary care, health outcomes or health inequalities and (5) used study designs that were either randomized control trials (RCTs) or quasi-experimental studies with controls.

Studies were excluded if they (1) assessed interventions at the community or village level and clinical trials; (2) did not have both temporal comparators and control groups; (3) examined outcomes which only included hospital care or did not measure the actual primary care delivery, such as using simulated clients and performance examinations for primary care providers and (4) were qualitative studies, commentaries, reviews, cross-sectional studies, uncontrolled before-after studies and uncontrolled interrupted time series studies.

One author performed the searches. The title and abstracts of all identified records were screened by two reviewers independently using Covidence (Veritas Health Innovation, 2021). Conflicts over study inclusion were discussed between the two reviewers and resolved by a third author. Full-text screening was undertaken by two reviewers independently, and disagreements were discussed and resolved with a third author. Data from eligible studies were extracted by the same two reviewers separately and cross-checked for errors. The quality of included studies was assessed by two reviewers.

### Data analysis

Information on the interventions, study settings, data sources, study designs, outcomes, effect directions, effect sizes, statistical significance and subgroup analysis was collected using a pre-designed standardized table. All relevant reported results were extracted. Outcomes were grouped into five categories: health expenditures (for both patients and providers), health service utilization, quality of care, health outcomes and health inequalities.

The quality of included studies was assessed using the Cochrane Collaboration’s tool for assessing Risk of Bias In Non-randomized Studies of Interventions (ROBINS-I) (Sterne *et al.*, 2016). Quality was assessed based on bias across seven domains: confounding, selection of participants into the study, classification of intervention (i.e. whether the interventions are clearly defined and the possibility of misclassification), deviation from intended interventions, missing data, measurement of outcomes and selection of the reported results. An overall risk of bias rating was generated. Each of the risk of bias domains was graded into one of the five categories: low, moderate, serious, critical and no information (Schünemann *et al.*, 2019). Compared with other quality assessment tools, ROBINS-I offers a comprehensive framework to identify weaknesses in intervention evaluations, but its reported risk of bias is often higher and with a smaller range of varieties (Losilla *et al.*, 2018) since only studies comparable to ‘a well-performed randomized trial’ can be judged as low risk of bias(Schünemann *et al.*, 2019). To address the limitation, the ROBINS-I tool in this review was adjusted whereby the quality of studies was upscaled one level if they balanced control groups using matching or weighting, included more than 4 years of observation, ruled out co-interventions or had random policy assignment (e.g. RCTs).

Heterogeneity in assessed policies, study populations and outcomes precluded meta-analysis, and a narrative approach was adopted. Studies were grouped by evaluated policies and outcome measures. A harvest plot was used to narratively summarize the heterogeneous effects of the PHC policies on each of the five outcome groups (Ogilvie *et al.*, 2008). Heterogeneous effects examined in the included studies were extracted without any pre-specification and were grouped into three types for the analysis based on what was identified in studies: (1) geographical; (2) socio-demographic; (3) people with and without non-communicable diseases (NCDs), including cardiovascular diseases, cancers, chronic respiratory diseases and diabetes (World Health Organization, 2022).

## Results

**Figure 1.**
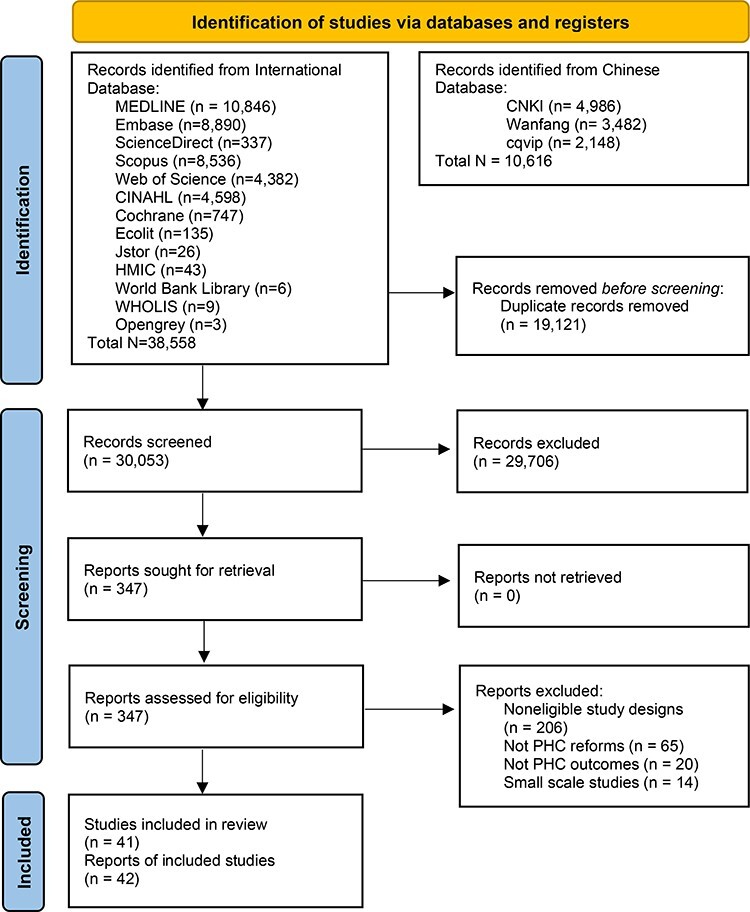
The Preferred Reporting Items for Systematic Reviews and Meta-Analyses diagram for study selection process

Notes: Small-scale studies were defined as studies that assessed interventions implemented within the community or village level.

The search identified 49 174 records. After removing duplicates, 30 053 studies were title and abstract screened and 347 studies were full-text screened (Figure 1). In total, 42 reports from 41 studies were eligible for inclusion, of which 31 were in English and 11 in Mandarin. Publication years ranged from 2012 to 2022. Eleven studies were nationwide, and 31 were based on cities/counties within provinces, except for two across provinces (Wei *et al.*, 2015; Yin *et al.*, 2016). There were 18 studies from eastern China, eight from central China (Yang *et al.*, 2013; 2017; Liu *et al.*, 2014; 2016; Jiang *et al.*, 2016; Miao *et al.*, 2018; 2019; Tang *et al.*, 2018) and five from western China(Powell-Jackson *et al.*, 2015; Tan *et al.*, 2015; Miao *et al.*, 2016; Yao *et al.*, 2020; Shen *et al.*, 2021). Nearly half (*N* = 18) studied rural populations, whilst 15 studies included both rural and urban populations. Eleven studies focused exclusively on people with NCDs, specifically with hypertension or diabetes. The study duration was <5 years for 36 studies, of which 13 had a duration of 2 years or less (Table 2).

**Table 2.** Characteristics of the 42 included studies

Characteristics of the included studies		Number of studies
Language	Mandarin	11
	English	31
Year of publication	2012–2015	17
	2016–2021	25
Geographical region	Central	8
	Eastern	18
	Western	5
	National	11
Study duration	Two years or less	13
	3–5 years	23
	More than 5 years	6
Data source	Primary data	7
	Secondary data	35
Data types	Cohort	8
	Panel	34
RCD data	No	12
	Yes	30
People with NCDs	No	31
	Yes	11
Urban/rural population	Rural	18
	Urban	9
	Both	15
Unit of analysis	Individual	22
	Aggregated	18
	Both	2
Evaluated policies	Comprehensive	5
	NEMP	15
	Financing	11
	System integration	4
	Gatekeeping	1
	Family physician	3
	NEPHS	1
	Workforce training	1
	Performance reporting	1
Study outcomes	Health expenditures	26
	Health service utilization	25
	Quality of care	13
	Health outcomes	10
	Health equity	1
Study quality	Low risk of bias	9
	Moderate risk of bias	24
	Serious risk of bias	9

All included studies were quasi-experimental studies, except for one matched-pair cluster RCT (Liu *et al.*, 2016). The majority of studies employed difference-in-difference (DID) approaches (*N* = 38), with seven combining this with propensity score matching/weighting (Zhang *et al.*, 2014a; 2017; Ding and Wu, 2015; Miao *et al.*, 2018; 2019; Hu *et al.*, 2021; Wang and Liu, 2022), eight using fixed-effect panel regression (Yi *et al.*, 2015; Sun *et al.*, 2016b; Yang *et al.*, 2017; Duan *et al.*, 2020; Xu *et al.*, 2020a; Shen *et al.*, 2021; Zhou *et al.*, 2021; Pan and Yang, 2022), three adopting dynamic DID models (also known as event-study analysis) (Yao *et al.*, 2020; Shen *et al.*, 2020a; Yuan *et al.*, 2021) and four without any statistical tests (Chen *et al.*, 2013; He *et al.*, 2014; Ma *et al.*, 2014; Wang *et al.*, 2014). Four studies were controlled interrupted time series studies (Yang *et al.*, 2013; Liang *et al.*, 2014; Sun *et al.*, 2016a; Tang *et al.*, 2018). Most studies used untreated populations in similar cities/counties in the province (*N* = 38), except for one based on populations from a different province (Yin *et al.*, 2016). Three studies used people with different intensities of the treatments (Yi *et al.*, 2015; Shen *et al.*, 2021) or alternative treatments (i.e. different combinations of provider payments and primary care delivery) across provinces (Wei *et al.*, 2015). There were 17 ecological studies at the facility level, whilst 22 studies used individual-level data. Most studies (*N* = 35) used secondary data, of which 30 used routinely collected data (RCD), such as administrative data from PHC facilities and health insurance data (Table 2).

**Figure 2.**
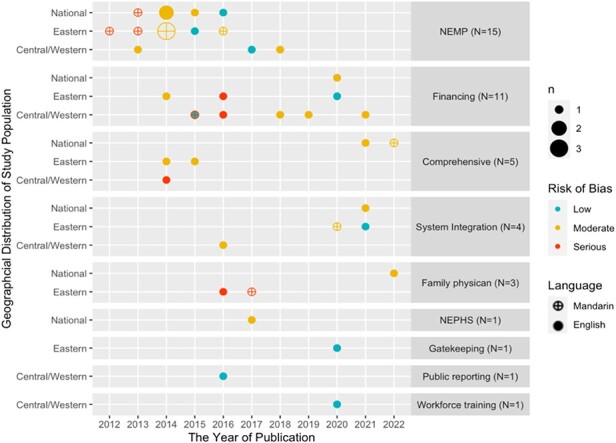
Language, publication year, geographical distribution and risk of bias of the 42 studies over time grouped by policies

Notes: *n* is the number of studies.

The 42 studies covered all the major PHC reforms, of which 37 assessed single-component reforms Of the five studies that evaluated the impacts of comprehensive PHC reform as a whole (Liang *et al.*, 2014; Liu *et al.*, 2014; Wei *et al.*, 2015; Zhou *et al.*, 2021; Pan and Yang, 2022), three focused on the first stage (2009–2014) (Liang *et al.*, 2014; Liu *et al.*, 2014; Wei *et al.*, 2015), whilst two examined the second stage (2015 onwards) (Zhou *et al.*, 2021; Pan and Yang, 2022). The NEMP was the most investigated PHC policy (*N* = 15), followed by financing reforms, Of the 11 studies on financing reforms, six focused on demand-side insurance reforms (e.g. reducing co-payment for visits to PHC facilities or NCD-related outpatient visits) (Zhang *et al.*, 2014a; Jiang *et al.*, 2016; Miao *et al.*, 2018; 2019; Shen *et al.*, 2020a; 2020b), two focused on provider payment reforms, such as P4P and capitation (Tan *et al.*, 2015; Sun *et al.*, 2016b), two focused on the combination of these two approaches (Powell-Jackson *et al.*, 2015; Sun *et al.*, 2016a) and one focused on governmental subsidies (Shen *et al.*, 2021). Of the five studies on reorganizing the health service delivery system, one assessed gatekeeping (Xu *et al.*, 2020a) and four examined the integrated delivery system (Miao *et al.*, 2016; Duan *et al.*, 2020; Hu *et al.*, 2021; Yuan *et al.*, 2021). Policies on primary care provision were the least studied, with three studies on the family physician policy (Wang and Liu, 2022; Yin *et al.*, 2016; Zhu *et al.*, 2017) and one study on the NEPHS (Zhang *et al.*, 2017). Additionally, two studies investigated two micro-level interventions on providers: (1) PHC workforce training (Yao *et al.*, 2020) and (2) public reporting on PHC facilities’ performance (Figure 2) (Liu *et al.*, 2016).

Health expenditures were the most studied outcome (26 studies). Three studies investigated out-of-pocket expenditures (OOPEs) (Ding and Wu, 2015; Miao *et al.*, 2018; 2019), whilst the rest examined NCD-related service or drugs costs in both PHC facilities and hospitals (*N* = 6) (Liu *et al.*, 2014; Zhang *et al.*, 2014a; Miao *et al.*, 2018; 2019; Shen *et al.*, 2020b; Hu *et al.*, 2021) or the costs in PHC facilities alone (*N* = 19). Health service utilization was examined by 25 studies, including visits to PHC facilities (*N* = 20), essential health service usage (*N* = 5) (Liu *et al.*, 2014; Wang *et al.*, 2014; Tan *et al.*, 2015; Yang *et al.*, 2017; Zhang *et al.*, 2017) and hospitalization (*N* = 9) (He *et al.*, 2014; Liu *et al.*, 2014; Powell-Jackson *et al.*, 2015; Yi *et al.*, 2015; Jiang *et al.*, 2016; Miao *et al.*, 2018; 2019; Shen *et al.*, 2020a; 2020b). Quality of care was examined by 13 studies from three aspects: antibiotic use (*N* = 8) (Jin *et al.*, 2013; Yang *et al.*, 2013; Chen *et al.*, 2014; Liang *et al.*, 2014; Gong *et al.*, 2016; Liu *et al.*, 2016; Sun *et al.*, 2016a; 2016b), satisfaction and perception of quality of care (*N* = 5) (Duan *et al.*, 2020; Liu *et al.*, 2014; Wei *et al.*, 2015; Yin *et al.*, 2016; Zhu *et al.*, 2017) and the delivery of essential medicines (*N* = 1) (Yang *et al.*, 2017). Ten studies investigated changes in health status using biomarkers (*N* = 8) (Duan *et al.*, 2020; Jiang *et al.*, 2016; Miao *et al.*, 2016; 2018; 2019; Zhang *et al.*, 2017; Hu *et al.*, 2021; Yuan *et al.*, 2021), mortality (*N* = 1) (Duan *et al.*, 2020), self-reported health status (*N* = 2) (Jiang *et al.*, 2016; Zhu *et al.*, 2017) and health-related quality of life (*N* = 2) (Miao *et al.*, 2016; Wang and Liu, 2022). Only one study assessed the reform effects on income-related health inequality (Pan and Yang, 2022) (Table 2). The effect sizes reported in the included studies were modest (Supplementary Appendix 3).

The risk of bias of the included studies ranged from low to serious, with nine studies assessed as high-quality (Ding and Wu, 2015; Powell-Jackson *et al.*, 2015; Gong *et al.*, 2016; Liu *et al.*, 2016; Yang *et al.*, 2017; Yao *et al.*, 2020; Shen *et al.*, 2020a; Xu *et al.*, 2020a; Hu *et al.*, 2021) and 24 with moderate risk of bias (Table 2). The most common potential bias was confounding from baseline differences and not accounting for concurrent interventions (*N* = 31) (Supplementary Appendix 2). The nine studies at serious risk of bias did not adjust for any potential confounders (Chen *et al.*, 2013; Jiang *et al.*, 2016; Jin *et al.*, 2013; Li *et al.*, 2012; Liu *et al.*, 2014; Sun *et al.*, 2016a; Tan *et al.*, 2015; Yin *et al.*, 2016; Zhu *et al.*, 2017), whilst one had a serious risk of bias from missing pre-intervention data and an inconsistent outcome measure (Sun *et al.*, 2016a). Missing data and deviation from the intended interventions were generally not discussed in the studies (14 and 23 out of 42, respectively) (Supplementary Appendix 2).

### Impacts of comprehensive PHC reforms

Of the five studies that examined the comprehensive reform as a whole package, three evaluated the first phase between 2009 and 2014 (Liang *et al.*, 2014; Liu *et al.*, 2014; Wei *et al.*, 2015) and two assessed the second phase since 2015 (Zhou *et al.*, 2021; Pan and Yang, 2022). There was no consensus on impacts, and the quality of evidence was low. One study in eastern China found drug expenditures and antibiotic use among children decreased following the 2009 reforms (Liang *et al.*, 2014), whilst a low-quality study from central China found that essential health service and hospital utilization and satisfaction increased, but outpatient visits (both at PHC facilities and hospitals) were unaffected (Liu *et al.*, 2014). A third study compared two PHC models introduced in 2009 in eastern China and reported that full government funding, better-trained health workforces and services tailored to local health needs were associated with a higher perceived quality of care (Wei *et al.*, 2015). Both studies on the second reform phase were nationwide and of moderate quality, with one study reporting an increase in primary care utilization among urban populations, but not their rural counterparts (Zhou *et al.*, 2021), with the other study showing that the reform decreased income-related health inequality (Table 3) (Pan and Yang, 2022).

**Table 3.** Estimated effects and certainty of evidence grouped by evaluated policies and outcomes

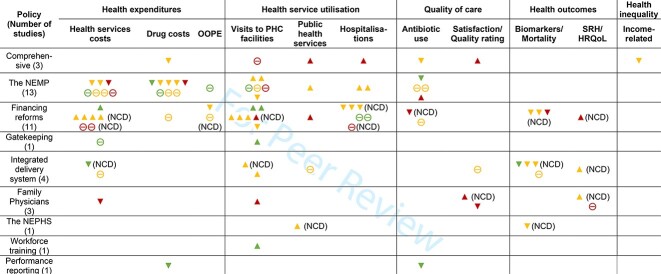

Notes: Each symbol represents one study on the corresponding assessed policies and outcomes, and studies assessing multiple outcomes have more than one symbol. NCD in brackets indicates that the study population were people with NCDs. ▲ indicates a statistically significant increase; ▼ indicates a statistically significant decrease; ⊝ indicates no statistically significant changes. Red indicates that the study was rated as serious risk of bias, yellow as moderate risk of bias and green as low risk of bias. Studies that only compared with alternative interventions or did not report overall impacts were not included in Table 3. Biomarkers including indicators from blood pressure and blood sugar tests and control rates. A decrease symbol in biomarkers indicates better hypertension/diabetes control.

Abbreviations: SRH, self-reported health; HRQoL, health-related quality of life.

### Impacts of the NEMP

There were 15 studies on the NEMP. Impacts were mixed, except for a consistent decrease in drug costs. Health outcomes were not studied. Seven ecological studies observed a reduction in costs for drugs at PHC facilities after NEMP introduction (Chen *et al.*, 2014; Gong *et al.*, 2016; Li *et al.*, 2012; Ma *et al.*, 2014; Tang *et al.*, 2018; Yang *et al.*, 2013; Zhang *et al.*, 2014b), but changes were insignificant in two national studies (Chen *et al.*, 2014; Zhang *et al.*, 2014b). One high-quality study on urban populations from eastern China found no impacts on drug costs or OOPE in PHC facilities (Ding and Wu, 2015). Three studies reported a decrease in healthcare costs in PHC facilities after the NEMP (Chen *et al.*, 2013; Han *et al.*, 2016; Ma *et al.*, 2014), whilst four studies found no impacts on healthcare costs (Ding and Wu, 2015; Li *et al.*, 2012; Yi *et al.*, 2015; Zhang *et al.*, 2014b). Of the six studies on health service utilization, two found an increase in the utilization of primary care services among rural populations in eastern China (He *et al.*, 2014; Han *et al.*, 2016), one study with no statistical test observed a decrease (Wang *et al.*, 2014) and three studies found no significant effects following NEMP introduction (Ding and Wu, 2015; Li *et al.*, 2012; Yi *et al.*, 2015), including one national study (Yi *et al.*, 2015). One study without statistical tests found that vaccination rates in rural areas increased (Wang *et al.*, 2014), whilst two studies found increasing inpatient visits in rural areas (He *et al.*, 2014; Yi *et al.*, 2015). Among the four studies on antibiotic use, one high-quality national study found decreased prescription of antibiotics in urban PHC facilities (Gong *et al.*, 2016), two intermediate quality studies found no significant impacts (Yang *et al.*, 2013; Chen *et al.*, 2014) and one low-quality study found an increase in antibiotic use (Jin *et al.*, 2013) after the NEMP (Table 3).

### Impacts of financing policies

The 11 studies on financing policies showed a general increase in primary care utilization across various regions and population groups, but the quality of evidence varied. Of the six studies on demand-side financing reforms, five were NCD-focused interventions (Zhang *et al.*, 2014a; Jiang *et al.*, 2016; Miao *et al.*, 2018; 2019; Shen *et al.*, 2020b) and one high-quality study assessed incentives for using PHC facilities (Shen *et al.*, 2020a). All the six studies found increasing charges for outpatient services provided by both hospitals and PHC facilities following a reduction in copayments (Zhang *et al.*, 2014a; Miao *et al.*, 2018; 2019; Shen *et al.*, 2020a; 2020b), except for one low-quality study (non-significant effects) (Jiang *et al.*, 2016). However, the six studies did not distinguish who covered these increased costs—either by insurers or individual out-of-pocket payments. Both studies on OOPE found decreased total OOPE after reducing copayments (Miao *et al.*, 2018; 2019), but with a significant decline only in inpatient OOPE (Miao *et al.*, 2018). All three studies on primary care utilization found increased visits to PHC facilities following co-payment reduction (Miao *et al.*, 2018; 2019; Shen *et al.*, 2020a). All five studies on hospitalizations showed decreased inpatient care visits after co-payment reductions for visits to PHC facilities (Miao *et al.*, 2018; 2019; Shen *et al.*, 2020a; 2020b), except for one low-quality study reporting no effects (Jiang *et al.*, 2016). The high-quality study further showed that outpatient visits to hospitals decreased, whilst primary care utilization increased (Shen *et al.*, 2020a). Three studies examining health outcomes were all based on people with NCDs and suggested that reducing co-payment was associated with decreased diastolic blood pressure (Miao *et al.*, 2018; 2019) and blood glucose (Jiang *et al.*, 2016) and increases in self-rated health (Jiang *et al.*, 2016) (Table 3). The health impacts of the demand-side financing reforms among the general population were not studied.

Four studies investigated the health system impacts of provider payment reforms (i.e. capitation or P4P) with (Powell-Jackson *et al.*, 2015; Sun *et al.*, 2016a) or without (Tan *et al.*, 2015; Sun *et al.*, 2016b) combining demand-side incentives. Three of the four studies found visits to PHC facilities (Powell-Jackson *et al.*, 2015; Tan *et al.*, 2015; Sun *et al.*, 2016a), and use of essential health services (Tan et al.) increased. No significant changes in inpatient care use were found (Powell-Jackson *et al.*, 2015). One high-quality study also found that a combination of both demand and supply-side financing reforms showed no greater effects on visits to PHC facilities than the sole demand-side insurance reform did (Powell-Jackson *et al.*, 2015). No studies found that provider payment reforms had significant effects on system costs (Sun *et al.*, 2016a; 2016b), antibiotic use (Sun *et al.*, 2016b) or intensity of treatment (Powell-Jackson *et al.*, 2015), except for one low-quality study reporting decreases in the use of both antibiotics and injections (Sun *et al.*, 2016a). One moderate-quality study using continuous DID showed that increasing government subsidies to PHC facilities had an adverse impact on visits to PHC facilities, suggesting that higher subsidies were insufficient to incentivize primary care practitioners to deliver additional services beyond the set goals (Shen *et al.*, 2021) (Table 3). The effects of provider payment reforms on health outcomes were not examined.

### Impacts of health service delivery policies

Regarding policies restructuring the service delivery system, one study assessed gatekeeping (Xu *et al.*, 2020a) and four examined integration of delivery systems (Miao *et al.*, 2016; Duan *et al.*, 2020; Hu *et al.*, 2021; Yuan *et al.*, 2021). The high-quality study on gatekeeping found that the use of primary care services increased, accompanied byb a decrease in outpatient visits to hospitals, but with no impact on health costs in PHC facilities (Xu *et al.*, 2020a). Of the four studies that examined integrated care, one high-quality study found reducing total health costs in all medical institutions (Hu *et al.*, 2021) and two moderate-quality found increased visits to PHC facilities (Duan *et al.*, 2020; Yuan *et al.*, 2021). All three studies were based on people with hypertension or diabetes and showed that service integration increased control rates for both the diseases (Hu *et al.*, 2021; Yuan *et al.*, 2021) and increased self-reported health (Miao *et al.*, 2016). One moderate-quality study on the general population found no significant impacts on essential health service use, health services costs, satisfaction, self-reported health or mortality (Duan *et al.*, 2020) (Table 3).

Four studies examined policies on primary care at the community level, including family physicians (Yin *et al.*, 2016; Zhu *et al.*, 2017; Wang and Liu, 2022) and the NEPHS (Zhang *et al.*, 2017). Two of the three studies on the family physician policy were low-quality and from eastern China (Yin *et al.*, 2016; Zhu *et al.*, 2017), whilst the other moderate-quality study used evidence from a national survey (Wang and Liu, 2022). One low-quality study using self-reported measures found reduced healthcare costs and satisfaction and increased visits to PHC facilities, but no changes in self-reported health (Zhu *et al.*, 2017). By contrast, two studies found improved perceived quality of care (Yin *et al.*, 2016) and health-related quality of life (Wang and Liu, 2022) among people with NCDs. One moderate-quality national study showed that the NEPHS increased uptake of antihypertensive drugs and blood pressure examinations among people with hypertension and increased blood pressure control rates (Zhang *et al.*, 2017) (Table 3).

The two micro-level provider interventions, workforce training (Yao *et al.*, 2020) and performance reporting (Liu *et al.*, 2016), were assessed by two high-quality studies, from western and central China, respectively. One found that PHC workforce training increased primary care utilization (Yao *et al.*, 2020), whilst one found that public reporting on the performance of PHC facilities reduced drug costs and the use of combined antibiotics (Liu *et al.*, 2016) (Table 3).

### Heterogeneous impacts of the reforms

**Figure 3.**
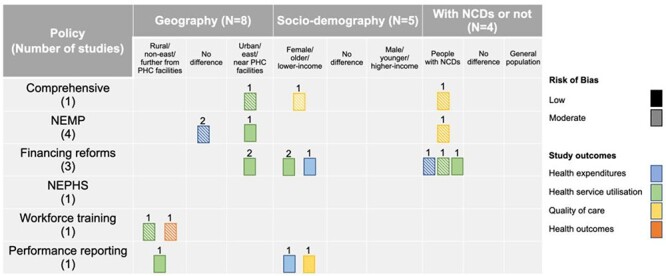
Heterogeneous effects of China’s PHC reforms

Notes: The matrix has six rows (one for each PHC policy) and three columns (one for each type of heterogeneities). A bar in the cell indicates that the included study found greater policy effects among the corresponding population groups (defined by the columns) compared to their counterfactuals. The bar in the columns of ‘No difference’ indicates that no differential policy effects were found. The number annotated on the top of each of the bars indicates the number of studies.

Heterogeneous impacts were investigated in 11 studies. Eight studies investigated differential effects across regions (i.e. urban/rural or eastern/western/central China) (Yang *et al.*, 2013; 2017; Chen *et al.*, 2014; Powell-Jackson *et al.*, 2015; Zhang *et al.*, 2017; Yao *et al.*, 2020; Shen *et al.*, 2020a; Zhou *et al.*, 2021). Two policies were found to benefit rural/western/central regions more in terms of health service utilization and health outcomes. The NEPHS was associated with larger increases in medication use, physical examinations and hypertension control in western/central China compared to eastern China (Zhang *et al.*, 2017). PHC workforce training increased visits to PHC facilities in rural regions but not in urban areas (Yao *et al.*, 2020). By contrast, the NEMP had no significant impacts on antibiotic use in either rural or urban areas (Yang *et al.*, 2013; Chen *et al.*, 2014), but reduced delivery rates of essential medicines in rural PHC facilities (Yang *et al.*, 2017). One study on the second stage of the PHC reform (Zhou *et al.*, 2021), as well as one on co-payment reductions (Shen *et al.*, 2020a), found increasing use of PHC facilities among urban areas but not rural areas. One study on financing reforms found that people living closer to village clinics had greater increases in clinic utilization (Powell-Jackson *et al.*, 2015) (Figure 3).

Among six studies examining heterogeneous reform impacts among people with different socio-demographic characteristics (Chen *et al.*, 2014; Powell-Jackson *et al.*, 2015; Wei *et al.*, 2015; Liu *et al.*, 2016; Shen *et al.*, 2020a; 2020b) and with/without NCDs(Chen *et al.*, 2014; Wei *et al.*, 2015; Shen *et al.*, 2020b), one from eastern China assessed the first stage of the comprehensive reform and found that people from poorer households or with NCDs had higher ratings of primary care quality after the reform (Wei *et al.*, 2015). Regarding single-component policies, four studies from various regions found that financing interventions disproportionally benefitted females (Powell-Jackson *et al.*, 2015; Shen *et al.*, 2020a), the elderly (Shen *et al.*, 2020a), people from poor households (Powell-Jackson *et al.*, 2015) and people with NCDs (Chen *et al.*, 2014; Shen *et al.*, 2020b) in terms of primary care utilization. One study on performance reporting also found greater improvement in rational use of health services among the elderly (Liu *et al.*, 2016) (Figure 3).

## Discussion

China’s PHC reforms since 2009 have made some progress towards the stated policy objectives of increasing primary care utilization and improving the health of people with NCDs. The PHC reforms disproportionally benefitted vulnerable populations (i.e. women, the elderly and lower-income populations) and under-resourced regions (i.e. rural areas and central/western China), suggesting some progress in reducing inequalities. However, the overall impacts were relatively modest. The reform impacts on care quality, financial protection and general population health were understudied, and evidence on healthcare system costs was mixed.

Comprehensive system-wide PHC reforms have been identified as a prerequisite for high-performing health systems and UHC achievement (Kruk *et al.*, 2018; Lancet Global Health, 2018; Kruk and Pate, 2020), yet few countries have introduced ambitious reforms such as those in China. Comprehensive PHC reforms that involve both demand and supply sides are more desirable than selective, single-component approaches to build strong health systems (Kruk *et al.*, 2018; Kruk and Pate, 2020) and achieve UHC (Atun, 2015) since single-component policies are unlikely to achieve system-wide changes or synergetic effects. Policy change across multiple health system functions aligns with and can accelerate progress to a ‘whole-of-society approach’ and multisector actions for health (World Health Organization, 2018). Additionally, the synergistic effects of comprehensive reforms can adapt to the dynamics and interconnected parts of health systems. Comprehensive PHC reforms have been implemented in a few middle-income countries, such as Brazil (Paim *et al.*, 2011), Mexico (González-Pier *et al.*, 2006; Knaul *et al.*, 2012), Bolivia (Alvarez *et al.*, 2016), Turkey (Atun *et al.*, 2013; Atun, 2015) and Iran (Heshmati and Joulaei, 2016; Ghasemyani *et al.*, 2022). These countries have well-established PHC sectors and share a paradigm of comprehensive PHC reforms—centring on family physician care models and financing reforms (Bitton *et al.*, 2019). Although China pioneered the ‘barefoot doctor programme’ in the 1960s to expand the coverage of primary care services in rural areas, this programme was unsuccessful in substantially changing the hospital domination of the health system due to scarce funds and unskilled workforces (Zhang and Unschuld, 2008; Xu *et al.*, 2020b). China’s hospital-centric health system remained dominated by large public hospitals (Liu *et al.*, 2017; Xu *et al.*, 2020b), with a drastically imbalanced health resource distribution and poor coordination between hospital and PHC sectors (Yip *et al.*, 2012). In this context, China’s PHC reforms have aimed to not only revive and strengthen the PHC system but also improve integration between the PHC and hospital sector, making it a valuable case study for other settings.

Existing evaluations of PHC reforms in LMICs have mainly focused on single-component PHC policies (Bastos *et al.*, 2017; Hone *et al.*, 2017; Angell *et al.*, 2019; Bitton *et al.*, 2019; Qin *et al.*, 2019); whilst there is a dearth of evidence on system-wide, comprehensive PHC reforms, Turkey’s comprehensive PHC reforms were found to increase infant and maternal health service utilization and improve health outcomes (Atun *et al.*, 2013). Similar impacts were found in Bolivia (Alvarez *et al.*, 2016) and other Latin American countries (Ramírez *et al.*, 2011). Comparably, this review found some evidence of increased use of maternal and general health services in China. However, there were no studies examining how comprehensive PHC reforms in the country affected health system costs, health outcomes and equity. The small number of studies and low-quality evidence identified in this review highlight a missed opportunity to examine the synergetic effects of China’s ambitious PHC reforms.

Regarding single-component PHC policies, our finding of increased primary care utilization in China aligns with evidence from reforms in other LMICs (Hone *et al.*, 2016; 2017; Qin *et al.*, 2019). This review found that deductible copayments for primary care services (Powell-Jackson *et al.*, 2015; Shen *et al.*, 2020a), free-of-charge essential public health services (Zhang *et al.*, 2017) and the introduction of family physicians (Zhu *et al.*, 2017) all increased primary care utilization in China. For people with hypertension or diabetes, these three interventions increased primary care service utilization and medication and treatment adherence, reduced hospitalizations and resultant OOPE (Zhang *et al.*, 2017; Miao *et al.*, 2018; 2019) and improved their health outcomes (Zhang *et al.*, 2017; Miao *et al.*, 2018; 2019; Wang and Liu, 2022). The management of other NCDs, such as cardiovascular diseases, was not investigated. There should be a cautious interpretation of these increases in primary care utilization. These increases might indicate increased access to care since unmet healthcare needs and undertreated NCDs are prevalent in China (Yip *et al.*, 2019; Wang *et al.*, 2021). It could also suggest a shift in patient flows from hospitals to PHC facilities—supported by two studies reporting that PHC increases were concomitant with reducing hospital utilization (Shen *et al.*, 2020a; Xu *et al.*, 2020a). However, the increases in primary care utilization could also be explained by overutilization as the appropriateness of this usage was not ascertained. Notably, short-term increases in PHC utilization following the reforms contradict with nationwide decreases in the preference for using PHC facilities (Ta *et al.*, 2020; Zhang *et al.*, 2020; Wan *et al.*, 2021) and the dwindling patient share of visits to PHC facilities (Liu *et al.*, 2021b). This contradiction suggests that the PHC reforms may have been ineffective in achieving enduring impacts, outpacing the growth of the hospital sector or reversing the hospital-centric orientation of the health system. Detailed understanding of the nature of primary care utilization, including appropriateness and efficiency, the long-term effectiveness of the reforms and relative improvement in the PHC system compared with the hospital sector is needed.

This review is unable to draw firm conclusions on reform impacts on service quality given that relevant evidence is scarce and of poor quality, and comprehensive measurements of care quality were missing. The mixed evidence on antibiotic use in this review, a useful indicator of quality, is consistent with a recent review from China (He *et al.*, 2019). Evidence on quality perception was mixed, and the reform impacts on process quality were not studied. This substantial evidence gap underscores the lack of knowledge on quality improvement and comprehensive quality-oriented performance measurements in China (Li *et al.*, 2020; Xiong *et al.*, 2022)—a common impediment in LMICs for the achievement of a high-performance PHC system and UHC (Lancet Global Health, 2018; Kruk and Pate, 2020).

The finding of health improvements in this review was concentrated in high-risk populations (i.e. people with NCDs), and the few studies on general population health identified in this review found no significant health impacts. Evidence from Latin America and other Asian countries showed general population health improvements identified following PHC reforms (Kruk *et al.*, 2010; Bastos *et al.*, 2017; Hone *et al.*, 2020). This difference may stem from three reasons. First, China’s PHC reforms prioritized NCD management (Xiong *et al.*, 2022), and some of the assessed co-payment reductions (Jiang *et al.*, 2016; Sun *et al.*, 2016a; Miao *et al.*, 2018; 2019; Shen *et al.*, 2020b) and service delivery models (Miao *et al.*, 2016) were targeted towards people with NCDs. This priority is reasonable since NCDs are poorly managed (Li *et al.*, 2020) and predicted to remain an obstacle in China’s path towards UHC (Liu *et al.*, 2021b). Second, this review highlights the scarce studies and lack of quality measurements of the health of general populations. Reported health outcomes were selective for hypertension and diabetes, and health improvements among general population may not be captured or have longer lag times to show. More sensitive measurements for population health (e.g. infant and maternal health, mental health and other cardiovascular diseases) were not assessed. Third, PHC policies that benefit the wider population may have been hampered by the high dependency on hospital sectors, the lack of system-level changes and the knowledge gaps on quality improvement mentioned earlier. China’s prioritization of people with NCDs aligns with the global commitment and efforts in NCD control (World Health Organization, 2022). Nevertheless, comprehensive system-wide PHC reforms remain imperative for LMICs to deliver benefits to wider populations and to achieve UHC (Kruk *et al.*, 2018; Kruk and Pate, 2020).

Impacts of China’s PHC reform on healthcare costs were unclear, with financial protection infrequently studied. This review finds that the NEMP reduced drug costs at PHC facilities, but did not affect OOPE—similar conclusions to a previous systematic review (Liu *et al.*, 2021a). The limited impacts on OOPE may stem from primary care providers compensating lost drug mark-up income with other health service charges (Ding and Wu, 2015), offsetting intended financial protection. This plausibly reflects an underfunded PHC system and potentially explains unchanged provider behaviours found in this review, such as continued overuse of antibiotics (Yang *et al.*, 2013; Chen *et al.*, 2014; Sun *et al.*, 2016b), overtreatment (Powell-Jackson *et al.*, 2015) and continued unnecessary hospitalization (Yi *et al.*, 2015). This finding hints at the interactions of financing with other health system building blocks and reiterates the necessity of a system perspective on PHC financing to curb OOPE (Hanson *et al.*, 2022).

The PHC reforms in China showed some pro-equity features, which is consistent with findings from other LMICs (Ramírez *et al.*, 2011; Atun *et al.*, 2013; Lankaran *et al.*, 2017) and the declining trend of inequalities in health service utilization in China (Liu *et al.*, 2021b). All assessed PHC policies disproportionally benefitted vulnerable populations, including the elderly, females, lower-income populations and high-risk populations (Chen *et al.*, 2014; Ding and Wu, 2015; Powell-Jackson *et al.*, 2015; Wei *et al.*, 2015; Liu *et al.*, 2016; Shen *et al.*, 2020a; 2020b). This finding is anticipated since China’s PHC reforms have aimed to narrow the gaps in health service accessibility between different population groups and regions to achieve UHC. Additionally, these population groups are more likely to use PHC facilities (Zhang *et al.*, 2020; Sang *et al.*, 2021; Tao *et al.*, 2021) and likely more responsive to improved access brought about by PHC reforms than their counterparts. Some evidence in this review shows that essential public health services and workforce training delivered more benefits to rural areas and middle/central China in terms of primary care utilization and health outcomes (Zhang *et al.*, 2017; Yao *et al.*, 2020), whilst financial interventions failed to do so. This may suggest that China’s PHC reforms in under-resourced regions faced challenges beyond affordability in increasing primary care utilization, such as low trust in primary care providers (Duckett *et al.*, 2016) and low accessibility of PHC facilities (Tao *et al.*, 2021). These challenges indicate the needs for improving the accessibility and quality of primary care services among under-resourced areas, which can be addressed by essential public health services and workforce training but not sole financing incentives.

This study has limitations. Our conclusions are constrained by the sparse evidence, varying study quality and the observational nature of studies. Heterogeneity across studies prevented meta-analysis. The exclusion of small-scale studies, including experimental studies, is a trade-off between internal and external validity of evidence and may overlook some relevant evidence. Well-conducted RCTs can produce robust causal inferences but may not produce generalizable knowledge needed to inform large-scale policies (Pritchett and Sandefur, 2015; Deaton and Cartwright, 2018). This review has key strengths. This is the first systematic review on the topic and addresses an evidence gap on the impact of comprehensive PHC reforms in Asia and globally (Macinko *et al.*, 2009; Bitton *et al.*, 2019). Comprehensive search strategies and broad search terms were used to minimize the possibility of omitting eligible studies. Studies were searched for in English and Mandarin. The review only focused on robust evidence by restricting eligible study designs to RCTs and controlled before-after studies. The evidence on China’s PHC reforms mirrors wider weaknesses in PHC research in LMICs (Adam *et al.*, 2011; Alvarez *et al.*, 2016; Bitton *et al.*, 2019). This includes considerable dearth of evaluations with rigorous study designs, inadequate data and limited measurement of quality of primary care and health outcomes, which hampers robust and insightful research. Evidence gaps in PHC research often overlap with policy gaps in PHC reforms and hinder effective evidence-based policy-making, creating a vicious cycle. The concentrated evidence on financing and primary care utilization found in this review aligns with the identified ‘major policy initiatives’ of China’s health reform, which were considerably strengthened over time (Xiong *et al.*, 2022). By contrast, the scarce evidence on care quality highlighted in this review resonates with the policy gaps in performance improvements (Xiong *et al.*, 2022; Li *et al.*, 2017). Most of the studies in this review did not discuss how well the reforms were implemented or deviations from the intended interventions. Due to the lack of detailed process evaluations, we cannot conclude whether the mixed results found in this review were due to poor design or poor implementation of the reforms Existing studies mostly focused on single-component PHC interventions, whereas synthesized evidence and evaluations on the comprehensive reforms as a whole are rare. Understanding of long-term health system and health impacts and financial protection remains limited. Policy makers and PHC researchers in LMICs should work together and closely to address these challenges in the future.

## Conclusion

China’s comprehensive PHC reform since 2009 has made some progress in increasing primary care utilization, improving equity and improving some health outcomes for people with hypertension or diabetes. Progress regarding other objectives of the reforms is not clear. A question remains as to whether China’s PHC reforms have been sufficiently wide-reaching and transformative to achieve a truly PHC centric healthcare system given evidence of continued dominance of the hospital sector (Liu *et al.*, 2021b). Whilst there remains a need to create a stronger knowledge base of comprehensive system-wide PHC reforms globally, including evaluations with more robust study designs and quality impacts, the mixed evidence from China shows that evidence-based comprehensive reforms and strong PHC systems are essential for altering hospital domination and achieving UHC, providing crucial lessons to inform and advance PHC policy initiatives globally.

## Supplementary Material

czad058_Supp

## Data Availability

All data collected and generated in this systematic review are available from the corresponding author upon request.

## Reflexivity statement

The authors include one female and five males and have various levels of seniority. The author group for this paper includes researchers from various institutions in both LMICs and high-income countries, including China, UK and Australia. All the authors have expertise in health system research and policy evaluations in LMICs with a focus on PHC.
