# Supplementary material for: Health and health system impacts of China’s comprehensive primary healthcare reforms: a systematic review
Source: Health Policy Plan. 2023 Jul 28;38(9):1064–78. doi: 10.1093/heapol/czad058 (PMC10566320; doi:10.1093/heapol/czad058)
Supplement: czad058_Supp [file czad058_supp.zip › suppl_data/Appendix_revised.pdf]

Appendix 1 Search strategy for MEDLINE and CNKI database

Database: Medline (via PubMed)

Domains	Number	Keywords
Primary health care	1	“primary health care”[Title/Abstract] OR “primary care”[Title/Abstract] OR “primary health*”[Title/Abstract] OR “primary medical care”[Title/Abstract] OR “PHC”[Title/Abstract] OR “primary health service*”[Title/Abstract] OR “primary level care”[Title/Abstract] OR “primary-level care”[Title/Abstract] OR “primary level facilit*”[Title/Abstract] OR “primary-level facilit*”[Title/Abstract] OR “primary practice*”[Title/Abstract] OR “community care” [Title/Abstract] OR “community health*” [Title/Abstract] OR “village clinic” [Title/Abstract] OR “township health*” [Title/Abstract] OR “outpatient care” [Title/Abstract] OR “out-patient care”[Title/Abstract] OR “outpatient*” [Title/Abstract] OR “home care” [Title/Abstract] OR “basic medical care” [Title/Abstract] OR “basic healthcare” [Title/Abstract] OR “basic health care” [Title/Abstract] OR “basic health service*” [Title/Abstract] OR “home healthcare”[Title/Abstract] OR “home care” [Title/Abstract] OR “home-care” [Title/Abstract] OR “home healthcare” [Title/Abstract] OR “home health care” [Title/Abstract] OR “ambulatory care” [Title/Abstract] OR “ambulatory medical*”[Title/Abstract] OR “ambulatory health*”[Title/Abstract] OR “ambulatory service*”[Title/Abstract] OR “ambulatory facilit*”[Title/Abstract] OR “ambulatory center*”[Title/Abstract] OR “ambulatory centre*”[Title/Abstract] OR “ambulatory patient*”[Title/Abstract] OR “ambulatory practice*”[Title/Abstract] OR “primary health care” [Mesh] OR "Ambulatory Care"[Mesh] OR "Community Health Nursing"[Mesh] OR "Ambulatory Care Facilities"[Mesh] OR "Primary Care Nursing"[Mesh] OR "Home Care Services"[Mesh] OR "Community Medicine"[Mesh] OR "Community Health Services"[Mesh] OR "Continuity of Patient Care"[Mesh]
General Practice	2	“family physician*” [Title/Abstract] OR “family practice”[Title/Abstract] OR “family care” [Title/Abstract] OR “family medic*” [Title/Abstract] OR “general practice*” [Title/Abstract] OR “general practitioner*”[Title/Abstract] OR “family doctor*” [Title/Abstract] OR “contract*” [Title/Abstract] OR “GP” [Title/Abstract] OR “GPs” [Title/Abstract] OR “usual source of care” [Title/Abstract] OR "Home Care Services"[Mesh] OR "General Practice"[Mesh] OR "General Practitioners"[Mesh] OR "Physicians, Primary Care"[Mesh] OR "Nurses, Community Health"[Mesh] OR "Family Nurse Practitioners"[Mesh] OR "Physicians, Family"[Mesh] OR "Family Practice"[Mesh]
Interventions	3	reform*[Title] OR evaluat*[Title] OR intervention*[Title] OR polic*[Title] OR pilot*[Title] OR project*[Title] OR program*[Title] OR strateg*[Title] OR scheme*[Title] OR impact*[Title] OR effect*[Title] OR assess*[Title] OR affect*[Title] OR influenc*[Title] OR plan*[Title] OR "new rural cooperative" [Title/Abstract] OR "cooperative medical scheme" [Title/Abstract] OR "NRCMS" [Title/Abstract] OR "NCMS" [Title/Abstract] OR "urban resident basic medical insurance" [Title/Abstract] OR "urban employee basic medical insurance scheme" [Title/Abstract] OR OR "UEBMI" [Title/Abstract] OR "URBMI" [Title/Abstract] OR "URRMI" OR OR "Health Policy"[Mesh] OR "Health Planning"[Mesh] OR "Health Care Reform"[Mesh] OR "Evaluation Studies as Topic"[Mesh] OR "Program Evaluation"[Mesh] OR "Health Care Quality, Access, and Evaluation"[Mesh]
Location	4	“China” [Title/Abstract] OR “People's Republic of China” [Title/Abstract] OR “PRC” [Title/Abstract] OR “Chinese” [Title/Abstract] OR "China"[Mesh]
Limits	5	Time: 2009/01/01 – the date of search
	6	1 OR 2
	7	6 AND 3

	8	7 AND 4
	9	8 AND 5

Database: CNKI

**Domains**	**Number**	**Keywords**
Primary health care	1*	TI = (初级医疗 + 初级保健 + 初级卫生 + 基层医疗 + 基层卫生 + 基层保健 + 社区医疗 + 社区卫生 + 乡村医疗 + 乡村卫生 + 乡镇卫生中心 + 乡镇卫生院 + 村卫生室 + 家庭医生 + 全科医生)
Referral system and gatekeeping	2	1* AND TI = (分级诊疗 + 分级医疗 + 双向转诊 + 基层首诊 + 社区首诊 + 门诊统筹 + 急慢分治 + 转诊 + 医疗资源下沉 + 分工) [done:354]
System integration	3	1* AND 医联体 + 医疗联合体 + 医共体 + 县域一体化 + 县域分级 + 对口支援 + 医改 + 改革
Financing	4	1* AND 经费 + 资金 + 财政 + 支出 + 收入 + 收支 + 补偿 + 拨款 + 拨付 + 预付 + 资源配置 + 医疗资源

Medication	5	1* AND 药品零差 + 药品零加成 + 药品加成 + 基本药物 + 基药 + 药品供应 + 药品定价 + 药政 + 药品管理 + 集中采购
Information	6	1* AND 健康档案 + 数据 + 信息系统 + 信息技术 + 电子病历 + 一卡通
Workforce	7	1* AND 人力资源 + 人员流动 + 薪酬 + 激励 + 职业发展 + 职业规划 + 培训 + 技能 + 人员能力 + 教育 + 轮转 + 绩效 + 工资制 + 聘用制
Insurance	8	1* AND 医保 + 保险 + 自付 + 个人筹资 + 总额预算 + 总额预付 + 条目预算 + 预付 + 后付 + 报销 + 新农合 + 新型农村合作医疗 + 封顶 + 起付 + 救助 + 临床路径 + 按人头 + 按项目支付 + 按病种 + 病历支付 + 支付方式 + 支付制度 + 支付体系 + 支付方法
Financial protection	9	1* AND 医疗费用 + 诊疗费用 + 费用负担 + 经济负担 + 医疗支出 + 医疗负担
Quality	10	1* AND 医疗质量 + 服务质量 + 患者满意 + 满意度 + 患者态度 + 用药合理性 + 合理用药 + 医疗安全 + 服务效率 + 依从性 + 循证 + 同质 + 医疗可及性 + 服务可及性 + 就医行为 + 医疗服务利用 + 医疗服务使用 + 以人为本 + 以病人为中心 + 连续性 + 持续性 + 连贯性 + 公平 + 平等
Health outcomes	11	1* AND 健康状况 + 自评健康 + 健康水平 + 人口健康 + 健康不平等 + 健康促进

Limits	12	Time: 2009/01/01 to the date of search
	13	2 AND 12
	14	3 AND 12
	15	4 AND 12
	16	5 AND 12
	17	6 AND 12
	18	7 AND 12
	19	8 AND 12
	20	9 AND 12
	21	10 AND 12
	22	11 AND 12

Appendix 2 Risk of bias assessment using ROBINS-I tool

Table B.1 A table for ROBIN-I quality assessment on the included 42 studies

Study No	Author and Year	Confounding	Selection of participants	Classification of interventions	Deviations from intended interventions	Missing data	Measurement of outcomes	Selection of the reported result	Overall
1	He, 2014[1]	Moderate - potential for co-intervention	Low	Low	No info	No info	Low	Low	Moderate
2	Zhu, 2017[2]	Serious - substantial baseline difference among participants in the two groups	Low	Low	No info	Low	Moderate - All outcome variables were self-reported, and participants were aware of the treatment	Low	Serious
3	Han, 2016[3]	Moderate - potential for baseline difference and co-intervention	Low	Low	No info	No info	Low	Low	Moderate
4	Ma, 2014[4]	Moderate - potential for co-intervention	Low	Low	No info	No info	Low	Low	Moderate
5	Wang, 2014[5]	Moderate - potential for baseline difference and co-intervention	Low	Low	No info	No info	Low	Low	Moderate
6	Chen, 2013[6]	Serious - baseline difference was not controlled and potential for co-intervention	Low	Low	No info	No info	Moderate - data were collected via institutional questionnaire and participated institutions were aware of the treatment.	Low	Serious
7	Li, 2012[7]	Serious - baseline difference was not controlled and potential for co-intervention	Low	Low	No info	No info	Low	Low	Serious
8	Duan, 2020[8]	Moderate - potential for baseline difference	Low	Low	No info	Moderate - the distribution of missing data was unclear	Low	Low	Moderate
9	Tan, 2015[9]	Serious - potential for baseline difference, which also affected treatment assignment.	Low	Low	No info	No info	Moderate - data were collected via questionnaire and participants were aware of the treatment.	Low	Serious
10	Jin, 2013[10]	Serious - baseline difference was not controlled	Low	Low	No info	Low	Low	Low	Serious
11	Chen, 2014[11]	Moderate - potential for co-intervention.	Low	Low	No info	Low	Low	Low	Moderate
12	Ding, 2015[12]	Low	Low	Low	No info	Low	Low	Low	Low
13	Gong, 2016[13]	Low	Low	Low	No info	No info	Low	Low	Low

Study No	Author and Year	Confounding	Selection of participants	Classification of interventions	Deviations from intended interventions	Missing data	Measurement of outcomes	Selection of the reported result	Overall
14	Jiang, 2016[14]	Serious - substantial difference at baseline characteristics and potential uncontrolled self-selection bias.	Low	Moderate - the treatment received or not and the time of receiving were self-reported	Low	Low	Moderate - Outcome were subjective and participants were aware of the treatment.	Low	Serious
15	Liang, 2014[15]	Moderate - baseline characteristics for the two groups were not described.	Low	Low	Low	Moderate - substantial missing data from pre-trending in the intervention group	Low	Moderate - The results for subgroups were not reported	Moderate
16	Liu, 2014[16]	Serious - substantial baseline difference across the groups. Failed to separate the time trend of the outcomes.	Low	Low	Low	Moderate - a substantial amount of data on satisfaction score were missing.	Moderate -Outcome were subjective and participants were aware of the treatment.	Moderate - the study did not clearly stage what outcome variables will be assess in the method session	Serious
17	Shen, 2020a[17]	Low	Low	Low	Low	Low	Low	Low	Low
18	Sun, 2016a[18]	Moderate - Potential for co-intervention.	Low	Low	Low	Serious - large number of cases with pre-intervention data were excluded	Serious - The measurements of outcome were inconsistent and pre- and post-intervention data were from data resources.	Low	Serious
19	Tang, 2018[19]	Moderate - Potential baseline confounding	Low	Low	Low	No info	Moderate - The outliners suggested potential measure bias.	Low	Moderate
20	Wei, 2015[20]	Moderate - Potential for baseline difference., and pre-intervention trend was not tested	Low	Low	No info	Low	Moderate - Outcome were subjective and participants were aware of the treatment.	Low	Moderate
21	Xu, 2020[21]	Low	Low	Low	Low	Low	Low	Low	Low
22	Yang, 2017[22]	Low	Low	Low	No info	Low	Low	Low	Low
23	Yang, 2013[23]	Moderate - potential bias on treatment assignment.	Low	Low	Low	No info	Low	Moderate - results from statistic tests were not presented	Moderate
24	Yao, 2020[24]	Low	Low	Low	Low	Low	Low	Low	Low
25	Yin, 2016[25]	Serious - substantial baseline difference. No control of time-varying confounding.	Low	Moderate - Based on the location of the participates, less accurate.	No info	Low	Moderate - Outcome were subjective and participants were aware of the treatment.	Low	Serious
26	Zhang, 2017[26]	Moderate - Potential for baseline difference.	Low	Moderate - Based on retrospective questions from the survey	No info	Low	Moderate - possible recall bias on medication uses and BP monitoring	Low	Moderate
27	Zhang, 2014a[27]	Moderate - Potential for baseline difference.	Low	Low	Low	No info	Low	Low	Moderate
28	Zhang, 2014b[28]	Moderate - Potential of co-intervention	Low	Low	Low	No info	Low	Low	Moderate

Study No	Author and Year	Confounding	Selection of participants	Classification of interventions	Deviations from intended interventions	Missing data	Measurement of outcomes	Selection of the reported result	Overall
29	Zhou, 2021[29]	Low	Low	Low	No info	Low	Moderate - Outcome were subjective and participants were aware of the treatment.	Low	Moderate
30	Miao, 2019[30]	Moderate - Outcome variable was used in PSM.	Low	Low	Low	Low	Low	Low	Moderate
31	Powell-Jackson, 2015[31]	Low	Low	Low	Low	Low	Low	Low	Low
32	Shen, 2020b[32]	Moderate - potential baseline difference	Low	Low	Low	No info	Low	Low	Moderate
33	Yi, 2015[33]	Low	Low	Low	No info	Moderate - substantial missing data	Moderate - the study reported falsified health service records.	Low	Moderate
34	Miao, 2018[34]	Moderate - potential baseline difference, time-varying confounding, and bias from co-intervention	Low	Low	Low	Low	Low	Low	Moderate
35	Sun, 2016b[35]	Moderate - potential baseline difference	Low	Low	Moderate – one treatment group switched to a treatment group different from the original plan	Moderate - substantial missing data on post-intervention periods	Moderate - Outcome were subjective and participants were aware of the treatment.	Low	Moderate
36	Miao, 2016[36]	Moderate - potential baseline difference	Low	Low	Low	Low	Moderate - Outcome were subjective and participants were aware of the treatment.	Low	Moderate
37	Liu,2016[37]	Low	Low	Low	Low	Low	Low	Low	Low
38	Hu, 2021[38]	Low	Low	Low	No info	Low	Low	Low	Low
39	Wang, 2022[39]	Moderate - potential bias from co-intervention	Low	Moderate – potential cut-off point inaccuracy	No info	Low	Low	Low	Moderate
40	Yuan, 2021[40]	Moderate - potential baseline difference	Low	Moderate - how the intervention was identified were not well-described	No info	Low	Low	Low	Moderate
41	Shen, 2021[41]	Moderate - potential bias from co-intervention	Low	Low	Low	Moderate - cases with missing values were excluded	Low	Low	Moderate
42	Pan, 2022[42]	Moderate - potential baseline difference	Low	Low	No info	No info	Low	Low	Moderate

Figure B.1 A traffic light plot for risk-of-bias assessments on the included 42 studies

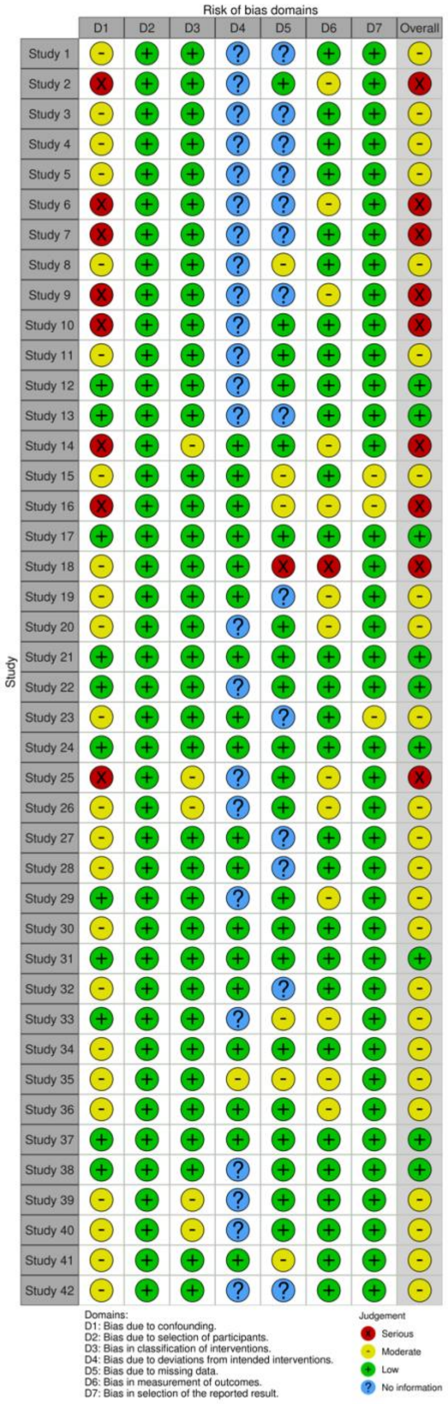

Figure B.2 A summary for risk-of-bias assessments on the included 42 studies

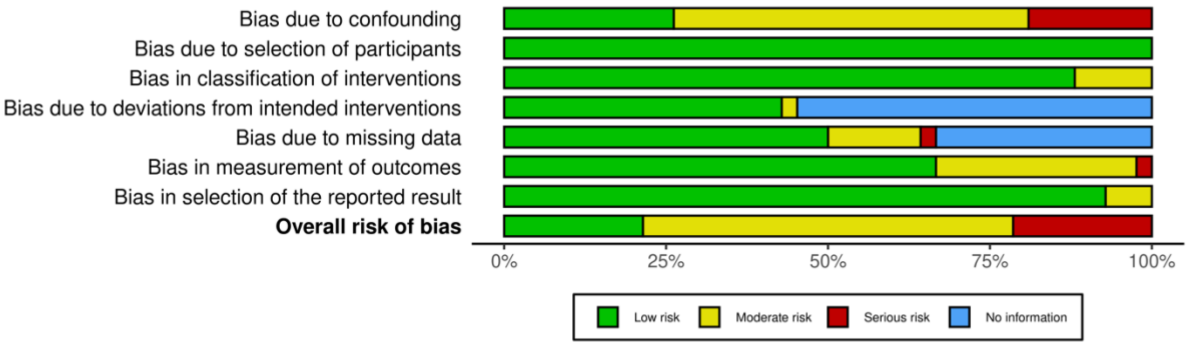

Appendix 3. Study characteristics and data extraction of the included 42 studies

Study characteristics of the included 42 studies

Study information			Data			Study population				Policy	Study design		
Author and Year	Duration	Language	Data source	Routinely collected data	Cohort/ Panel	Geography	Age	Urban/ Rural	With NCDs	Policy Label	Comparator	Statistic models	Unit of analysis
He, 2014[1]	2010-2011	CH	Secondary	Yes	Panel	Eastern Within province	NA	Rural	No	NEMP	No exposure	DID with no test	Facilities
Zhu, 2017[2]	2013, 2014	CH	Primary	No	Panel	Eastern Within province	All age	Urban	No	Family physician	No exposure	DID	Individual
Han, 2016[3]	2012-2013	CH	Secondary	Yes	Panel	Eastern Within province	NA	Rural	No	NEMP	No exposure	DID	Facilities
Ma, 2014[4]	2009-2010	CH	Secondary	Yes	Panel	Eastern Within province	NA	Both	No	NEMP	No exposure	DID with no test	Facilities
Wang, 2014[5]	2009-2010	CH	Secondary	Yes	Panel	Eastern Within province	NA	Rural	No	NEMP	No exposure	DID with no test	Facilities
Chen, 2013[6]	2007 and 2010	CH	Secondary	Yes	Panel	National	NA	Both	No	NEMP	No exposure	DID with no test	Facilities
Li, 2012[7]	2009-2010	CH	Secondary	Yes	Panel	Eastern Within province	NA	Rural	No	NEMP	No exposure	DID	Facilities
Duan, 2020[8]	2010-2014	CH	Secondary	Yes	Panel	Eastern Within province	NA	Urban	No	System Integration	No exposure	DID with FE	Facilities
Tan, 2015[9]	2011-2013	CH	Secondary	Yes	Panel	Western Within province	NA	Both	No	Financing	No exposure	DID	Facilities
Jin, 2013[10]	Eight months	CH	Secondary	Yes	Cohort	Eastern Within province	Mainly <18 (All age)	Rural	No	NEMP	No exposure	DID	Individual

Study information			Data			Study population				Policy	Study design		
Author and Year	Duration	Language	Data source	Routinely collected data	Cohort/ Panel	Geography	Age	Urban/ Rural	With NCDs	Policy Label	Comparator	Statistic models	Unit of analysis
Chen, 2014[11]	2007-2010	EN	Secondary	Yes	Panel	National	NA	Both	No	NEMP	No exposure	DID	Facilities
Ding, 2015[12]	2008-2010	EN	Secondary	Yes	Panel	Eastern<br>Within province	Age Mean= 58.7 (18+)	Urban	No	NEMP	No exposure	DID with PSM	Individual
Gong, 2016[13]	2007-2011	EN	Secondary	Yes	Panel	National	NA	Urban	No	NEMP	No exposure	DID	Facilities
Jiang, 2016[14]	one year	EN	Primary	No	Panel	Central<br>Within province	Aged 40–70 years	Rural	Yes	Financing	No exposure	DID	Individual
Liang, 2014[15]	2008-2010	EN	Secondary	Yes	Cohort	Eastern<br>Within province	Children younger than 5 years of age	Urban	No	Comprehensive	No exposure	ITS with control	group
Liu, 2014[16]	2009 and 2010	EN	Primary	No	Cohort	Central<br>Within province	Age Mean= 33.6 (All age)	Rural	No	Comprehensive	No exposure	DID	Individual
Shen, 2020a[17]	2013-2015	EN	Secondary	Yes	Panel	Eastern<br>Within province	17.78% 60+ (18+)	Urban	No	Financing	No exposure	Event study	Individual
Sun, 2016a[18]	2008-2012	EN	Secondary	Yes	Panel	Eastern<br>Within province	~ 80% 18–65 years (All age)	Both	No	Financing	No exposure	ITS with control	Both
Tang, 2018[19]	2011–2013	EN	Secondary	Yes	Panel	Central<br>Within province	NA	Both	No	NEMP	No exposure	ITS with control	Facilities
Wei, 2015[20]	2011, 2013	EN	Primary	No	Cohort	Eastern<br>Across province	Age ≥ 60 (18+)	Urban	No	Comprehensive	Alternative	DID	Individual

Study information			Data			Study population				Policy	Study design		
Author and Year	Duration	Language	Data source	Routinely collected data	Cohort/ Panel	Geography	Age	Urban/ Rural	With NCDs	Policy Label	Comparator	Statistic models	Unit of analysis
Xu, 2020[21]	2012-2014	EN	Secondary	Yes	Panel	Eastern<br>Within province	Age Mean= 46.9 (18+)	Rural	No	Gatekeeping	No exposure	DID with FE	Individual
Yang, 2017[22]	2011–2013	EN	Secondary	Yes	Panel	Central<br>Within province	NA	Both	No	NEMP	Alternative + no exposure	DID with FE	Facilities
Yang, 2013[23]	2009-2011	EN	Secondary	Yes	Panel	Central<br>Within province	NA	Both	No	NEMP	No exposure	ITS with control	Facilities
Yao, 2020[24]	2012–2016	EN	Secondary	Yes	Panel	Western<br>Within province	NA	Both	No	Workforce training	No exposure	Event study	Facilities
Yin, 2016[25]	2011, 2013	EN	Primary	No	Cohort	Eastern<br>Across province	Aged ≥18 years	Urban	Yes	Family physician	No exposure	DID	Individual
Zhang, 2017[26]	2011–2013	EN	Secondary	No	Panel	National	Adults 45 years and older	Both	Yes	NEPHS	No exposure	DID with PSW	Individual
Zhang, 2014a[27]	2010 and 2011	EN	Secondary	Yes	Panel	Eastern<br>Within province	~45% Age between 61 and 80 (All age)	Rural	Yes	Financing	Intensity + no exposure	DID with PSM	Individual
Zhang, 2014b[28]	2009 and 2010	EN	Secondary	Yes	Panel	National	NA	Rural	No	NEMP	No exposure	DID	Facilities
Zhou, 2021[29]	2012, 2014, 2016, 2018.	EN	Secondary	No	Panel	National	Age≥16 years	Both	No	Comprehensive	No exposure	DID with FE	Individual
Miao, 2019[30]	2015, 2017	EN	Secondary	Yes	Panel	Central<br>Within province	Age Mean = 65.0 ± 5.1(18+)	Rural	Yes	Financing	No exposure	DID with PSM	Individual

Study information			Data			Study population				Policy	Study design		
**Author and Year**	**Duration**	**Language**	**Data source**	**Routinely collected data**	**Cohort/ Panel**	**Geography**	**Age**	**Urban/ Rural**	**With NCDs**	**Policy Label**	**Comparator**	**Statistic models**	**Unit of analysis**
Powell-Jackson, 2015[31]	2009, 2011	EN	Primary	No	Panel	Western Within province	About 30	Rural	No	Financing	Alternative + no exposure	DID	Individual
Shen, 2020b[32]	2015-2016	EN	Secondary	Yes	Panel	National	above 18 (not specified in the study)	Urban	Yes	Financing	No exposure	DID	Individual
Yi, 2015[33]	2004, 2007, 2011	EN	Secondary	No	Cohort	National	Age Mean= 65.0 ± 5.1 (18+)	Rural	No	NEMP	Intensity	DID with FE	Both
Miao, 2018[34]	2015, 2017	EN	Secondary	Yes	Panel	Central Within province	Age Mean= 66.4 ± 7.9 (18+)	Rural	Yes	Financing	No exposure	DID with PSM	Individual
Sun, 2016b[35]	2011, 2012	EN	Secondary	Yes	Cohort	Eastern Within province	NA	Rural	No	Financing	No exposure	DID with FE	Individual
Miao, 2016[36]	2012, 2014	EN	Primary	No	Panel	Western Within province	Age Mean= 66.3 ± 10.7 (18+)	Rural	Yes	System Integration	No exposure	DID	Individual
Liu,2016 [37]	2012-2014	EN	Secondary	Yes	Cohort	Central Within province	Age Mean =37.96 (All age)	Rural	No	Public reporting	No exposure	DID	Individual
Hu, 2021[38]	2012-2016	EN	Secondary	Yes	Panel	Eastern Within province	Elderly	Both	Yes	System Integration	No exposure and alternative	DID with PSM	Individual
Wang, 2022[39]	2013,2015, 2018	EN	Secondary	No	Panel	National	Elderly	Both	Yes	Family physician	No exposure	DID with PSM	Individual

Study information			Data			Study population				Policy	Study design		
Author and Year	Duration	Language	Data source	Routinely collected data	Cohort/ Panel	Geography	Age	Urban/ Rural	With NCDs	Policy Label	Comparator	Statistic models	Unit of analysis
Yuan, 2021[40]	2009-2018	EN	Secondary	Yes	Panel	National	NA	Both	Yes	System Integration	Alternative + no exposure	DID with FE	Facilities
Shen, 2021[41]	2009-2017	EN	Secondary	Yes	Panel	Western<br>Within province	NA	Rural	No	Financing	percentage - continuous variables	DID with FE	Facilities
Pan, 2022[42]	2011-2018	CN	Secondary	No	Panel	National	Elderly	Both	No	Comprehensive	No exposure	DID with FE	Individual

Notes: DID for difference-in-differences models; DID with no test for studies that used difference-in-differences models but did not conduct any statistic tests; PSM for propensity score matching; PSW for propensity score weighting; ITS for interrupted time series analysis; FE for fixed effects with multiple time points. NA for not applicable.

Estimated policy effects in the included 42 studies

Author and Year	Estimated effects
He, 2014[1]	Health service utilisation:<br>The number of visits to PHC facilities (visits): DID= 4055.43<br>The number of inpatient care visits (visits): DID= 1061.43<br>Notes: no statistic tests were done.
Zhu, 2017[2]	Perceptions/awareness:<br>Policy awareness rates (%): (DID= 0.063, P<0.001)<br><br>Health care costs:<br>Agreement on family physician policy reducing health care costs (%): (DID= 0.21, P=0.099)<br><br>Satisfaction:<br>Supportive attitude on first-contact with PHC facilities (DID= -0.04, P=0.025)<br><br>Health service utilization:<br>Self-reported first contact with community health stations (DID= 0.159, P<0.001)
Han, 2016[3]	Health expenditure:<br>Expenditures for outpatient visits to PHC facilities per capita (yuan) DID= -30.350 (P< 0.001)<br><br>Health service utilization:<br>The number of outpatient care visits in PHC facilities per month (visits) DID= 135.067 (P=0. 017)
Ma, 2014[4]	Health expenditure:<br>Health service costs for outpatient care per visits(providers) (yuan): DID= -61.37<br>Medical expenditures for outpatient care per visits (patients) (yuan): DID= 3.12<br>Drug costs per outpatient care visits (patients) (yuan): DID= -64.48<br>Notes: no statistic tests were conducted.
Wang, 2014[5]	Health service utilization:<br>Total number of outpatients visits to PHC facilities per year (visits): DID = -1902.00,<br>Total number of vaccines per year (visits): 1448.00;<br>Notes: no statistic tests were conducted.
Chen, 2013[6]	Health expenditure:<br>Medical expenditures per visit to PHC facilities (yuan): DID= -10.3<br>Notes: no statistic tests were conducted.
Li, 2012[7]	Health service utilization:<br>Total number of outpatient visits to PHC facilities (visits): DID= -1165.59(P=0.82)<br>Total number of inpatient visits to PHC facilities (visits): DID= -26.78 (P= 0.92)<br><br>Health expenditure:<br>Drug expenditures for outpatient care per capita (yuan): DID= -7.11(P=0.04)<br>Medical expenditures for outpatient care per capita (yuan): DID= 0.79 (P=0.76)<br>Drug expenditures for inpatient care per capita (yuan): DID= -227.20 (P=0.02)<br>Medical expenditures for inpatient care per capita (yuan): DID= -6.86 (P=0.94)
Duan, 2020[8]	Health service utilisation:<br>The number of people under diabetes management [43]:-0.086(0.054)<br>The number of people under hypertension management [43]:-0.071(0.062)

Author and Year	Estimated effects
	Elderly health management:-0.049(0.056)<br>Total number of visits to PHC facilities per capita (visits): 0.362***(0.133)<br><br><u>Health expenditure</u><br>Health care costs for outpatient visits to PHC facilities per visits (yuan):-9.705(8.233)<br><br><u>Health outcomes</u><br>Diabetes incidence rates(%): 0.002(0.006)<br>Hypertension incidence rates(%): -0.020(0.014)<br>The number of deaths:1.490(11.820)<br><br><u>Quality of care:</u><br>Patient satisfaction (%): 0.002(0.024)<br>Notes: Coefficient (SE), *, ** and *** for significance at p <= 0.1, 0.05, 0.001, log for log-transformed outcomes
Tan, 2015[9]	<u>Perceptions/awareness:</u><br>Policy awareness rates (%): DID= 8.5(SE=3.77 , P=0.039)<br>Type 2 diabetes/ hypertension awareness rates(%): DID = 8.97 (SE= 1.8, P= 0.001)<br><br><u>Health service utilization:</u><br>Prenatal checkup rates(%): DID= 10.25(SE=5.09 , P=0.040)<br>Postnatal visit rates(%): DID= 12.09(SE= 5.66, P=0.048)<br>Health examination rates among 0-6 years old children(%): DID= 9.84(SE= 9.79, P=0.330)<br>Health examination rates among people with NCDs(%): DID= 6.55(SE=2.92 , P=0.001)<br>Health examination rates among elderly people aged 60 or over(%): DID= 12.36(SE=3.84 , P=0.005)<br>Vaccination rates among 0-6 years old children(%): DID= 6.49(SE=3.38 , P=0.073)<br>Examination rates among people with serious psychiatric illness (%): DID= 13.69(SE=5.75 , P=0.030)
Jin, 2013[10]	<u>Quality of care:</u><br>Antibiotic use - Whether there is a joint use of antibiotics, steroids, and intravenous infusion.<br>DID odd ratio= 2.46**(P<0.001)<br>DID Risk ratio= 2.27** (P<0.001) – The treatment groups had 1.27 times higher chance of the joint use.
Chen, 2014[11]	<u>Health service costs:</u><br>Urban: Average prescription expenditure [43] (yuan): DID= -0.31, SE=0.55<br>Rural: Average prescription expenditure [43] (yuan): DID= -0.21, SE=0.21<br><br><u>Quality of care:</u><br>Urban: Proportion of prescriptions contained two or more antibiotics (%): DID= 4.56, SE=9.48<br>Rural: Proportion of prescriptions contained two or more antibiotics (%): DID= -6.60, SE=5.09
Ding, 2015[12]	<u>Health service utilisation:</u><br>The number of annual outpatient visits (visits): DID=0.010, RSE= 0.027, P= 0.707<br>The number of annual outpatient visits in PHCs (visits): DID= 0.019, RSE=0.032 , P= 0.533<br><br><u>Health expenditures:</u><br>The total annual health care expenditure per capita [43] ($): DID=0.042 , RSE= 0.042 , P= 0.331<br>The annual health care expenditure per capita in PHCs [43] ($): DID=0.038 , RSE=0.048 , P= 0.446<br>The annual drug expenditure per capita [43] ($): DID= 0.087 , RSE=0.056 , P= 0.138

Author and Year	Estimated effects
	The annual drug expenditure per capita in PHCs [43] ($): DID=0.062 , RSE=0.071 , P= 0.395<br>The annual OOP expenditure per capita ($): DID= 0.065, RSE= 0.035 , P= 0.074<br>The annual OOP expenditure per capita in PHCs ($): DID=0.068, RSE= 0.043 , P= 0.133<br>Notes: RSE for robust standard error. Only results after propensity score matching were included.
Gong, 2016[13]	<u>Quality of care:</u><br>Percentage of prescription with antibiotics: -7(2)***(95% CI: -10, -4);<br>Percentage of prescription with >1 antibiotics: -2(1)**(95% CI: -3, 0);<br>Percentage of prescription with injection: -2(2);<br><br><u>Health expenditures:</u><br>Expenditures per prescription (yuan): -8(3)***(95% CI: -14, -2)<br>Notes: coefficients (SE), *P<0.10; **P<0.05; ***P<0.01.
Jiang, 2016[14]	<u>Health service utilisation:</u><br>Seen at county or higher level hospital (binary): base line OR=0.78 (P=0.56, 95% CI: 0.35–1.76), follow-up OR= 0.77 (P=0.85, 95% CI: 0.34–1.72);<br>Number of hospital visits*:* OR=1.01 (P=0.70 ,95% CI:0.95–1.07)<br><br><u>Health outcomes:</u><br>SF–12 Physical: OR=1.06 (P= 0.01 ,95 % CI: 1.01–1.10)<br>SF–12 Mental: OR=1.01 (P=0.40 ,95 % CI:0.98–1.05)<br>EQ–5D Health scale: OR= 0.97 (P=0.06 ,95 % CI: 0.94–1.00)<br>The haemoglobin A1c (HbA1c): OR= 0.74 (P=0.02 95 % CI: 0.56–0.99)<br><br><u>Health expenditures:</u><br>The feeling of burdensome (binary): baseline OR=1.37 (P=0.36, 95 % CI: 0.64–2.92), follow-up OR=0.83 (P=0.90, 95 % CI: 0.37–1.83);
Liang, 2014[15]	<u>Quality of care:</u><br>Proportion of patients receiving antibiotic use per month (%): Group* level change −0.0434; Group* trend change −0.0103<br>Proportion of patients receiving two or more antibiotics per month (%): Group* level change −0.0734*; Group* trend change −0.0084<br><br><u>Health expenditures:</u><br>Average monthly costs of antibiotics per patient [43] (yuan): Group* level change 0.1101; Group* trend change −0.1377*<br>Average monthly cost of medications per patient [43] (yuan): Group* level change 0.0867; Group* trend change −0.0477*
Liu, 2014[16]	<u>Health service utilisation:</u><br>Outpatient attendance rates in past 2 weeks (‰): Treated group: in 2009: 61.3 (55.1 to 67.4) in 2010: 59.5 (52.6 to 67.0); Control group: in 2009:37.6 (29.7 to 45.5), in 2010: 51.4 (42.4 to 60.4)<br>Hospitalisation rates (‰): Treated group: in 2009: 28.2 (24.0 to 32.5), in 2010: 58.0 (50.7 to 65.3)*; Control group: in 2009: 79.8 (78.1 to 81.4), in 2010: 87.3 (75.8 to 98.8)<br>Antenatal exam rates in the past year (%): Treated group: in 2009: 69.0%, (65.8 to 73.1), in 2010: 75.8%, (72.2 to 79.4) Control group: in 2009: 62.4%, (56.0 to 68.8), in 2010: 57.6%, (50.3 to 64.9)<br>Postpartum visit rates (%): Treated group in 2009: 37.2%, (32.6 to 41.8), in 2010: 29.9, (23.8 to 36.0)**; Control group: in 2009: 23.9, (14.8 to 33.0), in 2010: 26.9 (17.9 to 36.4)<br>Health examinations rates among respondents 60 years and older (%): Treated group: in 2009: 65.5 (61.5 to 69.5), in 2010: 89.7 (86.9 to 92.5)**; Control group: in 2009: 38.0 (32.4 to 43.6) 2010: 54.1 (47.5 to 60.7)**<br><br><u>Quality of care:</u><br>Satisfaction score: Treated group 2009: 21.4 (21.1 to 21.7) 2010: 22.1 (21.7 to 22.4) t= 2.754; P= 0.006; Control group 2009:20.6 (20.1 to 21.1) 2010: 20.2 (19.7 to 20.8) t=0.943 ; P= 0.347<br>Notes: 95% CI were shown in the parathesis. ** for P<0.001, * for P<0.05.

Author and Year	Estimated effects
Shen, 2020a[17]	Health service utilisation:<br>Total number of outpatient visits (visits): 0.018*** (0.004)<br>Number of outpatient visits at primary care providers (visits): 0.036*** (0.003)<br>Number of outpatient visits at non-primary care providers (visits): −0.018*** (0.003)<br>Number of visits for overall care, including hospitalisation (visits): −0.004 (0.050)<br>Hospitalisation days for overall care, including hospitalisation (days): −0.004 (0.050)<br>Hospitalisation days for ambulatory care sensitive conditions (ACSCs) (days): 0.123 (0.113)<br>Number of visits for ACSCs (visits): 0.003 (0.021)<br><br>Health expenditures:<br>Total health care spending per month (yuan): −0.0001 (0.013)<br>Spending per month at primary care providers (yuan): 0.132*** (0.009)<br>Spending per month at non-primary care providers (yuan): −0.106*** (0.011)<br>Spending per visit for overall care (yuan): 0.001 (0.007)<br>Spending per visit for ACSCs (yuan): 0.022 (0.228)<br>*Notes: Standard errors were shown in parentheses. ***p < .01. **p < .05. *p < .10.*
Sun, 2016a[18]	Healthcare utilization:<br>Number of OP visits to PHC facilities per month (visits): Level change: 46 895 (0.004, 15 795~77 994); Trend change: 673 (0.74, -3453~4799)<br><br>Health expenditure:<br>Total expenditures per visit (yuan): Level change: -15.40 (0.16, -36.95~6.15); Trend change: -0.81 (0.48, -3.10~1.47)<br><br>Quality of care:<br>The percentage of visits with at least one antibiotic (%): Level change: -1.11 (0.76, -8.34~6.11); Trend change: 1.52 (0.02, 0.21~2.83)<br>The percentage of visits with at least one injectable (%): Level change: -7.38 (0.03, -14.08~-0.68); Trend change: -0.92 (0.06, -1.89~0.05)<br>Notes: Coefficient (P value, 95% CI)
Tang, 2018[19]	Health expenditures:<br>Total cost for antibiotics per month (million yuan): Level change: − 8.31, [− 18.68, 2.06], P= 0.114; Trend changes − 1.56 [− 2.96, − 0.15], P= 0.030<br><br>Quality of care:<br>Total defined daily dose for antibiotics per month (in million): level changes: − 0.32 [− 3.92, 3.27], P= 0.857; Trend changes − 0.51 [− 0.97, − 0.05], P= 0.031<br>Notes: Coefficient [95% CI]
Wei, 2015[20]	Quality of care:<br>*First contact:* Utilization: DID= 0.255 (0.176 to 0.333); Accessibility DID= −0.030 (−0.082 to 0.022)<br>*Continuity of care:* DID= 0.233 (0.166 to 0.300)<br>*Coordination:* Services DID= 0.083 (−0.009 to 0.175); Information DID= 0.233 (0.147 to 0.320)<br>*Comprehensiveness:* Service availability DID= 0.325 (0.262 to 0.388); Service provided DID= 0.454 (0.362 to 0.546)<br>*Patient-focused care:* DID= 0.098 (−0.027 to 0.222)<br>*Overall quality score:* DID= 1.651 (1.266 to 2.037)<br>Notes: Coefficient (95% CI).
Xu, 2020[21]	Health service utilisation:<br>Average ambulatory care visits to PHC facilities per enrolee per quarter: DID= 0.142, 95% CI= (0.071 to 0.212), P=0.001<br>Average ambulatory care visits to hospitals per enrolee per quarter: DID= −0.026, 95% CI= (−0.051 to 0.001), P= 0.040

Author and Year	Estimated effects
	Health expenditure:<br>Total ambulatory care expenditure to PHC facilities per enrolee per quarter (yuan): DID= 0.840, 95% CI= (−11.126 to 12.806), P=0.884<br>Total ambulatory care expenditure to hospitals per enrolee per quarter (yuan): DID= −22.600, 95% CI= (−139.167 to 6.034), P= 0.011
Yang, 2017[22]	Health service utilisation:<br>The delivery rates of essential medicines in urban areas (%):<br>Medicine-tied model: DID=6.32, P=0.10, 95%CI= −1.15, 13.80<br>The delivery rates of essential medicines in rural areas (%):<br>Medicine-tied model: DID=−7.78, P=0.00, 95%CI= −11.25, −4.31<br>Recipient-tied model: DID=−19.85, P=0.00, 95%CI= −24.85, −14.84<br>Recipient-medicine-tied model DID=3.99, P=0.24, 95%CI= −2.67, 10.65
Yang, 2013[23]	Health expenditures:<br>Average expenditure per prescription (yuan): level change= -18 (p<0.03)<br><br>Quality of care:<br>Percentage of prescriptions requiring antibiotics (%): non-significant change, the values were not reported.<br>Percentage of prescriptions requiring injections (%): non-significant change, the values were not reported
Yao, 2020[24]	Health service utilisation:<br>*Rural:*<br>Total annual number of visits to PHC facilities (visits) in rural areas: Coefficient = 0.105* (1.973)<br>Annual number of outpatient visits to PHC facilities (visits) in rural areas: Coefficient = 0.141** (2.196)<br>The share of total visits to PHC facilities among all medical institutions in rural areas (%): Coefficient = 0.0395** (2.429)<br>*Urban:*<br>Total annual number of visits to PHC facilities in urban areas: -0.0914 (-0.914)<br>Annual number of outpatient visits to PHC facilities (visits) in urban areas: -0.122 (-1.172)<br>The share of total Visits to PHC facilities among all medical institutions in urban areas (%): -0.0160 (-0.630)<br>Notes: T-statistics were shown in the parathesis. *** p<0.01, ** p<0.05, * p<0.1
Yin, 2016[25]	Quality of care:<br>First contact utilization: DID= 0.21 (0.11, 0.31)***; First-contact accessibility DID= 0.04 (−0.05, 0.12)<br>Continuity of care DID= 0.04 (−0.06, 0.14)<br>Coordination of services: DID= 0.27 (0.12, 0.42)***; Coordination of information: DID= 0.10 (0.01, 0.20)*<br>Comprehensiveness service availability: DID= 0.01 (−0.08, 0.10); Comprehensiveness service provided: DID= 0.06 (−0.01, 0.12)<br>Family centeredness: DID= −0.04 (−0.17, 0.09)<br>Community orientation: DID= 0.18 (0.06, 0.31)**<br>Cultural competence: DID= 0.44 (0.25, 0.62)***<br>Total scores: DID= 1.30 (0.74, 1.87)***<br>Notes: Coefficients (95% CI), *P < 0.05; **P < 0.01; ***P < 0.001.
Zhang, 2017[26]	Health outcomes:<br>Hypertension control rate (%): 8.1* (3.4)<br><br>Health service utilisation:<br>Medication use rate (%): 9.1* (3.8)<br>Blood pressure monitoring rate (%): 9.5* (3.8)<br>Notes: Coefficients (SE), *P < 0.05; **P < 0.01; ***P < 0.001. Results after propensity-score weighting were included.
Zhang, 2014a[27]	Health service utilisation:<br>Ratio of visits by outpatients to different health institutions<br>Village clinic: DID = −0.434 t-value=−0.29

Author and Year	Estimated effects
	Township health centres: DID =5.985*** t-value= 4.55<br>County-level hospital: DID = 2.341*** t-value= 1.97<br><br>Health expenditure:<br>Annual total outpatient medical expenditure [43]:<br>When yearly maximum reimbursement amount increased by 40 yuan: DID =0.087***, t-value= 3.13<br>When yearly maximum reimbursement amount increased by 100 yuan: DID =0.157*** , t-value= 4.71<br>Notes: * P < 0.1; **P< 0.05; ***P< 0.01.
Zhang, 2014b[28]	Health expenditure:<br>Average drug expenditures for outpatients [43]: DID: −0.119 (0.058) FE: −0.123 (0.062)<br>Average drug expenditures for inpatients [43]: DID: −0.266** (0.067) FE: −0.258** (0.064)<br>Average inpatient medical expenditures per capita: DID: −0.113* (0.041) FE: −0.106* (0.036)<br>Average outpatient medical expenditures per visit: DID: −0.042 (0.044) FE: −0.043 (0.042)<br><br>Health service utilisation:<br>Average number of inpatients stay per certified doctor per day: DID= 0.213 (0.184), FE: 0.171 (0.165)<br>Average number of visits per certified doctor per day: DID= −1.262 (1.129), FE: −1.152 (0.962)<br>Notes: FE for results from fixed effect models; Robust SEs in parentheses; *P<0.05, **P<0.01.
Zhou, 2021[29]	Health service utilisation:<br>Whether respondents usually go to PHC facilities to seek health services when they are sick? (Binary):<br>Urban OR = 1.261 (95% CI= 1.013 to 1.571, P < 0.05),<br>Rural OR = 0.894 (95% CI= 0.734 to 1.090, P = 0.269)<br>Whether respondents usually go to PHC facilities to seek health services when they are sick? (2 years after HMS implementation):<br>Urban OR= 1.498 (95% CI= 1.068 to 2.102, P < 0.05)<br>Rural: OR= 0.968 (95% CI=0.712 to 1.317, P = 0.836)
Miao, 2019[30]	Health Expenditure:<br>Per capita annual total outpatient expenditure (yuan): 81.2*, relative effects= 31.8%<br>Per capita annual total inpatient expenditure (yuan): –475.4*, relative effects= –40.7%<br>Per capita annual total expenditure (yuan): –394.2*, relative effects= –27.7%<br>Per capita annual total out-of-pocket expenditure (yuan): –201.9*, relative effects= –29.9%<br><br>Health service utilisation:<br>Per capita outpatient visits to PHC facilities (visits): 3.3**, relative effects= 81.0%<br>Per capita annual inpatient visits to PHC facilities (visits): –0.075**, relative effects= –60.0%<br>Per capita annual visits to PHC facilities (visits): 3.225**, relative effects= 76.8%<br><br>Health outcomes:<br>Diastolic blood pressure (mmHg): –2.9*; Systolic blood pressure (mmHg): –7.9.<br>The prevalence of complications increased slightly during this 1-year intervention in general, but the difference was not statistically significant (p>.05).<br>Notes: *P<0.05, **P<0.01, ***P<0.001. Information on CIs or SEs were not reported.
Powell-Jackson, 2015[31]	Health service utilisation:<br>Doctor visits in past 2 weeks: Benefit package: 0.015 (0.037); Benefit package + provider incentives: 0.036 (0.038)<br>Self-treated or no treatment sought: Benefit package: -0.015 (0.037); Benefit package + provider incentives: -0.036 (0.038)<br>Outpatient visits to at village clinics: Benefit package: 0.053 (0.026)**; Benefit package + provider incentives: 0.060 (0.030)**<br>Outpatient visits to township health centres: Benefit package: -0.001 (0.024); Benefit package + provider incentives: 0.021 (0.022)

Author and Year	Estimated effects
	Outpatient visits to country hospitals: Benefit package: 0.010 (0.020); Benefit package + provider incentives: -0.008 (0.024)<br>Outpatient visits to provincial hospitals: Benefit package: -0.031(0.015)**; Benefit package + provider incentives: -0.007(0.013)<br>Inpatient care in past year (any): Benefit package: -0.004 (0.006); Benefit package + provider incentives:0.003 (0.005)<br>Inpatient care at township health centre (any): Benefit package:0.000 (0.003); Benefit package + provider incentives: 0.002 (0.003)<br>Inpatient care at county hospital (any): Benefit package: -0.001 (0.004); Benefit package + provider incentives: 0.002 (0.004)<br>Inpatient care at provincial hospital (any): Benefit package: -0.003 (0.003); Benefit package + provider incentives:-0.001 (0.002)<br>Number of admissions in past year: Benefit package: -0.003 (0.009); Benefit package + provider incentives: 0.008 (0.009)<br><br>The intensity of treatment:<br>Injection during outpatient visits in the past 2 weeks (any): Benefit package: 0.116 (0.039)***; Benefit package + provider incentives: 0.090 (0.044)**<br>IV drip during outpatient visits in the past 2 weeks (any) : Benefit package: 0.116 (0.051)**; Benefit package + provider incentives: 0.053 (0.045)<br>Notes: Robust standard errors clustered at the village level are reported in parentheses. *P<0.1, **P<0.05, ***P<0.01. Intervention type 1 only introduced benefit package, whilst the type 2 was a combination of benefit package and provider incentives.
Shen, 2020b[32]	Health expenditures:<br>Outpatient care spendings among people with hypertension only [43]: DID MD= -9.0 (-11.8, -6.1), P< 0.0001<br>Outpatient care spending among people with diabetes only [43]: DID MD= -8.9 (-15.7, -2.1), P= 0.01<br>Outpatient care spending among people with both hypertension and diabetes [43]: DID MD= -7.5 (-12.0, -3.0), P= 0.001<br><br>Health service utilisation:<br>Monthly hospitalization rate among people with diabetes only: DID OR= 0.962 (0.691, 1.341), P= 0.82<br>Monthly hospitalization rate among people with hypertension only: DID OR= 1.047 (0.901, 1.218), P= 0.54<br>Monthly hospitalization rate among people with both hypertension and diabetes: DID MD= 0.891 (0.743, 1.068), P= 0.21<br>*Notes: No Overall impacts were reported. Standard errors are clustered at the individual level.*
Yi, 2015[33]	Health service utilisation:<br>Number of annual inpatient visits to township health centres (THCs) [43]: DID= 1.27* (0.05)<br>Number of annual outpatient visits to THCs [43]: DID= -0.19 (0.52)<br>Probability of seeking outpatient services at THC for most recent episode of illness: DID= 0.02 (0.47)<br>Probability of seeking inpatient services at THC for most recent of episode of illness: DID= -0.00 (0.74)<br><br>Health expenditures:<br>Medical inpatient expenditure at THCs [43]: DID= -0.05 (0.89)<br>Medical outpatient expenditures at THCs among those not referred elsewhere [43]: DID= -0.09 (0.70)<br>*Notes: p-values were shown in the parenthesis; *** p<0.01, ** p<0.05, * p<0.1.*
Miao, 2018[34]	Health service utilisation:<br>Per capita annual outpatient care visits (visits): DID= 3.3 (P=0.008), relative effects= 81.0%<br>Per capita annual inpatient care visits (visits): DID= −0.075 (P=0.000), relative effects= − 60.0%<br>Total number of visits for medical services (visits): DID= 3.225 (P=0.001), relative effects= 76.8%<br><br>Health expenditures:<br>Per capita annual medical expenditures for outpatient care (yuan): DID=81.2 (P=0.048), relative effects= 31.8%<br>Per capita annual medical expenditures for inpatient care (yuan): DID=−475.4 (P=0.027), relative effects= − 40.7%<br>Total annual medical expenditures per cap: DID=−394.2 (P=0.013), relative effects= −27.7%<br>Patient per capita annual out-of-pocket expenditures for outpatient care (yuan): DID=−9.1 (P=0.518)<br>Patient per capita annual out-of-pocket expenditures for inpatient care (yuan): DID=−192.8 (P=0.027), relative effects = − 36.7%<br>Patient per capita annual out-of-pocket expenditures for total medical care (yuan): DID=−201.9 (P=0.022), relative effects= − 29.9%

Author and Year	Estimated effects
	Health Outcomes:<br>Systolic blood pressure: DID=−2.9 (P=0.011)<br>Diastolic blood pressure: DID=− 7.9 (P=0.508)
Sun, 2016b[35]	Health expenditures:<br>Total costs for consultation and medicines at township health centres (yuan): -7.001; relative effects (%) = -0.160; (SE= 10.582; P= 0.561)<br>Total costs for consultation and medicines at village clinics (yuan): -3.158; relative effects (%) = -0.107; (SE= 4.694; P= 0.535)<br><br>Quality of care:<br>Whether a prescription contained antibiotic at township health centres (%): -0.050; relative effects (%) = -0.105; (SE= 0.058; P=0.356)<br>Whether a prescription contained 2 or more antibiotics at township health centres: -0.076; relative effects (%) = -0.511; (SE= 0.040; P= 0.074)<br>Whether a prescription contained intravenous injection at township health centres: -0.096; relative effects (%) = -0.269; (SE= 0.075; P= 0.194)<br>Whether a prescription contained antibiotic at village clinics: -0.033; relative effects (%) = -0.056; (SE= 0.074; P= 0.635)<br>Whether a prescription contained 2 or more antibiotics at village clinics: 0.055; relative effects (%) = 0.485; (SE= 0.049; P= 0.304)<br>Whether a prescription contained intravenous injection at village clinics : -0.066; relative effects (%) = -0.184; (SE= 0.060; P= 0.284)<br>Notes: SE were adjusted for clustering at the township level. Adjustment for small number of clusters using wild bootstrapping.
Miao, 2016[36]	Health outcomes:<br>Health-related quality of life SF-36 *scale* scores: DID = 4.591; SE =1.794; P =0.011<br>Blood pressure (mm Hg): systolic blood pressure: DID = −5.62; SE =16.49; P =0.019, Diastolic blood pressure: DID = −5.43; SE =15.07; P =0.028
Liu,2016[37]	Quality of care:<br>Whether a prescription requires antibiotics or not (%): DID OR = 1.089 (1.067,1.110); P <0.001<br>Whether a prescription requires combined antibiotics or not (%): DID OR = 0.870 (0.850,0.890); P <0.001<br>Whether a prescription requires injections (%): DID OR = 1.258 (1.234,1.283); P <0.001<br><br>Health expenditures:<br>Average drug expenditures per prescription [43] (yuan): DID = -0.051 (-0.057, -0.045); P <0.001<br>Notes: 95%CI were shown in the parathesis.
Hu, 2021[38]	Health expenditures:<br>The total treatment costs per capita (yuan): hypertension group: DID= −380.530*** (16.632), diabetes groups: DID= −1117.247*** (54.917)<br><br>Health outcomes:<br>Control rates (%): hypertension: DID OR=1.407*** (0.007), Diabetes: DID OR= 1.333*** (0.011)<br>Notes: SEs were shown in parathesis. * P< 0.1 ** P< 0.05 *** P< 0.01
Wang, 2022[39]	Health outcomes:<br>Health-related quality of life scores: DID= 14.10 (SE = 0.044), P<0.05
Yuan, 2021[40]	Health service utilisation:<br>*Type 1 intervention - loose collaboration*<br>The number of annual outpatient visits to PHC facilities [43]: 0.120 (-0.027, 0.266)<br>The number of annual admissions in PHC facilities [43]: 0.251 (-0.134, 0.636)<br>The number of annual outpatient visits to hospitals: -0.043 (-0.220, 0.134)<br>The number of annual admissions in hospitals: -0.070 (-0.182, 0.042)<br>The ratio of outpatient visits between PHC facilities and hospitals (%): 3.456 (-1.241, 8.154)<br>The ratio of admissions between PHC facilities and hospitals (%): 3.269 (-1.373, 7.911)<br>*Type 2 intervention - tight integration*<br>The number of annual outpatient visits to PHC facilities [43]: 0.187 (-0.228, 0.601)<br>The number of annual admissions in PHC facilities [43]: 0.808 (0.503, 1.113)***<br>The number of annual outpatient visits to hospitals: -0.095 (-0.398, 0.209)

Author and Year	Estimated effects
	The number of annual admissions in hospitals: -0.074 (-0.197, 0.048)<br>The ration of outpatient visits between PHC facilities and hospitals (%): 4.855 (-2.241, 11.950)<br>The ratio of admissions between PHC facilities and hospitals (%): 9.263 (2.257, 16.270)*<br><br><u>Health outcomes:</u><br>*Type 1 intervention - loose collaboration*<br>Control rates of hypertension (%): 12.970 (2.816, 23.124)*<br>Control rates of diabetes (%):14.018 (5.837, 22.199)**<br>*Type 2 intervention - tight integration*<br>Control rates of hypertension (%): 24.699 (16.560, 32.838)***<br>Control rates of diabetes (%):17.655 (6.559, 28.752)**<br>Notes: 95% CI were shown in the parathesis. *** p<0.001, ** p<0.01, * p<0.05.
Shen, 2021[41]	<u>Health service utilisation:</u><br>The number of outpatient visit [43]: DID= −0.012*** (p< 0.001).
Pan, 2022[42]	<u>Health equity:</u><br>Centred index to measure income-related health inequality: DID = - 0.0192*** (t= -7.2457), p = 0.001

Notes: SE for standard error; CI for confident interval; log for log-transformed; DID for difference-in-differences.

Appendix 4 List of excluded studies and exclusion reasons

The 305 excluded studies after full-text screening and reasons

No	Excluded studies	Reasons for exclusion
1	Feng X, Feng W, Shen P, Wang Z, Shen J, Wang B. The effect of the integrated delivery system on managing hypertension in rural areas of China. Ann Palliat Med. 2021;10(1):434-42.	Noneligible study design
2	Guo Z, Guan X, Shi L. The impacts of implementation of National Essential Medicines Policies on primary healthcare institutions: a cross-sectional study in China. BMC Health Serv Res. 2017;17(1):723.	Noneligible study design
3	He J, Chen D, Tang Z, Cong L, Tian Y, Xie C, et al. How the health institution combinative contracting mechanism influences community residents' patient experiences in Shanghai: A comparative study of data from two cross-sectional surveys. Int J Health Plann Manage. 2019;34(3):1036-54.	Noneligible study design
4	Hu S. Essential medicine policy in China: pros and cons. J Med Econ. 2013;16(2):289-94.	Noneligible study design
5	Hu XY, Yang XJ. Early Intervention Practices in China Present Situation and Future Directions. Infant Young Child. 2013;26(1):4-16.	Noneligible study design
6	Hu Y, Luo S, Tang X, Lou L, Chen Y, Guo J, et al. Does introducing an immunization package of services for migrant children improve the coverage, service quality and understanding? An evidence from an intervention study among 1548 migrant children in eastern China. BMC Public Health. 2015;15:664.	Noneligible study design
7	Huang J, Lu W, Wang L, Zhang T, Liu C, Liu S, et al. A preliminary effect analysis of family doctor and medical insurance payment coordination reform in Changning District of Shanghai, China. BMC Fam Pract. 2019;20(1):60.	Noneligible study design
8	Huang J, Zhang T, Wang L, Guo D, Liu S, Lu W, et al. The effect of family doctor-contracted services on noncommunicable disease self-management in Shanghai, China. Int J Health Plann Manage. 2019;34(3):935-46.	Noneligible study design

9	Jiang B, Li YC, Zhang M, Huang ZJ, Liu Y, Wang LM. Expenditure in outpatient department and pharmacy on patients with hypertension and the influence from community health management program. Zhonghua Liu Xing Bing Xue Za Zhi. 2016;37(2):248-53.	Noneligible study design
10	Jin Y, Yuan B, Zhu W, Zhang Y, Xu L, Meng Q. The interaction effect of health insurance reimbursement and health workforce on health care-seeking behaviour in China. Int J Health Plann Manage. 2019;34(3):900-11."	Noneligible study design
11	Jin Y, Zhu W, Yuan B, Meng Q. Impact of health workforce availability on health care seeking behavior of patients with diabetes mellitus in China. Int J Equity Health. 2017;16(1):80.	Noneligible study design
12	Leng Y, Liu W, Xiao N, Li Y, Deng J. The impact of policy on the intangible service efficiency of the primary health care institution- based on China's health care reform policy in 2009. Int J Equity Health. 2019;18(1):14.	Noneligible study design
13	Li H, Gong Y, Han J, Zhang S, Chen S, Xu X, et al. Interrupted Time-Series Analysis to Evaluate the Impact of a National Antimicrobial Stewardship Campaign on Antibiotic Prescribing: A Typical Practice in China's Primary Care. Clin Infect Dis. 2020.	Noneligible study design
14	Li H, Wu Z, Hui X, Hu Y. Impact of local health insurance schemes on primary care management and control of hypertension: a cross-sectional study in Shenzhen, China. BMJ Open. 2019;9(10):e031098.	Noneligible study design
15	Li H, Zhu W, Xia H, Wang X, Mao C. Cross-Sectional Study on the Management and Control of Hypertension Among Migrants in Primary Care: What Is the Impact of Segmented Health Insurance Schemes? J Am Heart Assoc. 2019;8(16):e012674.	Noneligible study design
16	Li N, Du X, Dong J. Effect of Healthcare Reform on the Preferred Health Facility for Initial Consultation for Common Medical Symptoms among Residents in Beijing's Yuetan Area. Chinese General Practice. 2018;21(32):3991-3.	Noneligible study design
17	Li Q, Chen F, Yang M, Lu L, Pan J, Li X, et al. The Effect of China's National Essential Medicine Policy on Health Expenses: Evidence From a National Study. Inquiry. 2018;55:46958018787057.	Noneligible study design
18	Li SK, Zhang DL, Chen Z. Impact of the New Cooperative Medical Scheme on Individual Healthcare Utilization and Expenditure. Chin Econ. 2019;52(6):488-504.	Noneligible study design
19	Li Y, Ying C, Sufang G, Brant P, Bin L, Hipgrave D. Evaluation, in three provinces, of the introduction and impact of China's National Essential Medicines Scheme. Bull World Health Organ. 2013;91(3):184-94.	Noneligible study design
20	Li Z, Shu D, Xia M, Gao D, Lu D, Huang N, et al. The assessment on impact of essential drugs policy on primary health care system in rural areas of Shandong Province policy and regulation division of the Health Department of Shandong Province. Technol Health Care. 2015;23 Suppl 1:S169-76.	Noneligible study design

21	Li Z, Yang J, Li BY, Zhang L. The effects of tiered healthcare service delivery on the cost control and quality improvement in rural China: an interrupted time series analysis. Int J Integr Care. 2018;18(s1):1-2.	Noneligible study design
22	Liang C, Mei J, Liang Y, Hu R, Li L, Kuang L. The effects of gatekeeping on the quality of primary care in Guangdong Province, China: a cross-sectional study using primary care assessment tool-adult edition. BMC Fam Pract. 2019;20(1):93.	Noneligible study design
23	Liang ML, Liang CB, Li GL. Utilization Effect of Community First Contact Care System Among Medically Insured Residents in Chang'an Town of Dongguan and Influencing Factors. Chinese General Practice. 2016;19(16):1928-32.	Noneligible study design
24	Liu C, Zhang X, Wan J. Public reporting influences antibiotic and injection prescription in primary care: a segmented regression analysis. J Eval Clin Pract. 2015;21(4):597-603.	Noneligible study design
25	Liu GE, Guan HJ. Hierarchical Diagnosis and Treatment and General Practice Clinics: Key of China's Health Supply-side Reform. Chinese General Practice. 2016;19(22):2619-24.	Noneligible study design
26	Liu L, Fu X, Hu Y, Zhang B. Medical Insurance Payment Reform of Chongqing Medical Consortium. Chinese General Practice. 2020;23(7):795-8.	Noneligible study design
27	Liu P, Guo W, Liu H, Hua W, Xiong L. The integration of urban and rural medical insurance to reduce the rural medical burden in China: a case study of a county in Baoji City. BMC Health Serv Res. 2018;18(1):796.	Noneligible study design
28	Liu R, Sun Y, Jin L. Analysis of status of family doctor contracted services in Longbai community of Shanghai city and its influencing factors. Chinese Nursing Research. 2014;28(12B):4460-1.	Noneligible study design
29	Liu SS, Ge M, Jiang P, Zhu MJ, Liang H, Huang JL, et al. Effects of Contractual Services from Family Doctors on the Healthcare-seeking Behavior among Community Residents. Chinese General Practice. 2018;21(4):407-10.	Noneligible study design
30	Liu X, Hou Z, Towne SD, Jr., He M, Tan A, Jiang D, et al. Knowledge, attitudes, and practices related to the establishment of the National Hierarchical Medical System (NHMS) among outpatients in Chinese tertiary hospitals. Medicine (Baltimore). 2018;97(35):e11836.	Noneligible study design
31	Liu X, Sun X, Zhao Y, Meng Q. Financial protection of rural health insurance for patients with hypertension and diabetes: repeated cross-sectional surveys in rural China. BMC Health Serv Res. 2016;16(1):481.	Noneligible study design
32	Liu Y, Zhang JL, Cheng TCE, Ru YH, Hua GW. The Impacts of Drug Price Regulations in China. J Syst Sci Syst Eng. 2019;28(6):674-93.	Noneligible study design
33	Liu Z, Buijsen M. Legal reflections on the evolving role of general practitioners in China's primary care: an assessment of regulatory strategies. Prim Health Care Res Dev. 2019;20:e9.	Noneligible study design

34	Lu P, Zhu J, Jin M, Weng L, Lu Y. Development and Effectiveness Analysis of a Community-based Family Doctor-centered Care Delivery Model. Chinese General Practice. 2018;21(28):3430-5.	Noneligible study design
35	Ma J, Xu J, Zhang Z, Wang J. New cooperative medical scheme decreased financial burden but expanded the gap of income-related inequity: evidence from three provinces in rural China. Int J Equity Health. 2016;15:72.	Noneligible study design
36	Mao W, Huang Y, Chen W. An analysis on rational use and affordability of medicine after the implementation of National Essential Medicines Policy and Zero Mark-up Policy in Hangzhou, China. PLoS One. 2019;14(3):e0213638.	Noneligible study design
37	Minghui R, Guoping L. China's global health strategy. Lancet. 2014;384(9945):719-21.	Noneligible study design
38	Muennig P. What China's experiment in community building can tell us about tackling health disparities: community building and mental health in mid-life and older life: evidence from China. Soc Sci Med. 2014;107:217-20.	Noneligible study design
39	Pu X, Huang T, Wang X, Gu Y. Realigning the provider payment system for primary health care: a pilot study in a rural county of Zhejiang Province, China. Prim Health Care Res Dev. 2020;21:e43.	Noneligible study design
40	Qin JM, Lin CM, Zhang LF, Zhang YC. Patient Satisfaction with Primary Care in Highly Focused Districts/Counties during the Comprehensive Reform of Primary Care System in China. Chinese General Practice. 2018;21(1):36-40.	Noneligible study design
41	Qin JM, Lin CM, Zhang YC, Zhang LF. Construction of Primary Care Delivery System in Highly Focused Districts/Counties during the Comprehensive Reform of Primary Care System in China. Chinese General Practice. 2018;21(1):24-7.	Noneligible study design
42	Qin JM, Zhang LF, Lin CM. Scale and Allocation of Human Resources in Primary Health Care System in China After New Medical Reform. Chinese General Practice. 2016;19(4):378-82.	Noneligible study design
43	Ren J, Huang X, Zhang T, Zhou X, Liu C, Wang X. Patient satisfaction with prescribed medicines in community health services in China: A cross-sectional survey 6 years after the implementation of the national essential medicines policy. Health Soc Care Community. 2018;26(4):495-506.	Noneligible study design
44	Shi L, Makinen M, Lee DC, Kidane R, Blanchet N, Liang H, et al. Integrated care delivery and health care seeking by chronically-ill patients - a case-control study of rural Henan province, China. Int J Equity Health. 2015;14:98.	Noneligible study design
45	Shu Z, Wang L, Sun X. An evaluation of the effects of general practitioner-supported patient noncommunicable diseases control model in Shanghai, China. Int J Health Plann Manage. 2019;34(3):947-59.	Noneligible study design

46	Si X, Zhai Y, Zhu XL, Ma JX. The changing trend of capacity on policy implementation related to the prevention and control of chronic non-communicable disease at the provincial level, from 2011 to 2017. Zhonghua Liu Xing Bing Xue Za Zhi. 2019;40(6):726-30.	Noneligible study design
47	Song Y, Bian Y, Petzold M, Li L, Yin A. Effects of the National Essential Medicine System in reducing drug prices: an empirical study in four Chinese provinces. J Pharm Policy Pract. 2014;7(1):12.	Noneligible study design
48	Song Y, Bian Y, Petzold M, Li L, Yin A. The impact of China's national essential medicine system on improving rational drug use in primary health care facilities: an empirical study in four provinces. BMC Health Serv Res. 2014;14:507.	Noneligible study design
49	Song Y, Bian Y, Zhen T. Making medicines more accessible in China: An empirical study investigating the early progress of essential medicine system. PLoS One. 2018;13(8):e0201582.	Noneligible study design
50	Sun J, Luo H. Evaluation on equality and efficiency of health resources allocation and health services utilization in China. Int J Equity Health. 2017;16(1):127.	Noneligible study design
51	Sun MP, Rasooly A, Jian WY. Quality of primary health care in China: an analysis of data from a nationwide longitudinal survey. Lancet. 2018;392:74-.	Noneligible study design
52	Tang GB, Lin MQ, Li WH. Exploration and Evaluation of Hierarchical Diagnosis and Treatment of "Xiamen Mode". Chinese General Practice. 2016;19(22):2624-7.	Noneligible study design
53	Tang Q, Song P, Xu L. The role of family physicians contracted healthcare in China: A "Cardiotonic" or a "Band-Aid" for healthcare reform? Biosci Trends. 2016;10(4):325-6.	Noneligible study design
54	Tao ZW, Zhang ZY, Wang XL, Shi YQ, Soar J. Comparison of subsidy schemes for reducing waiting time: special focus on smart home care for elderly people. Proc Cirp. 2019;83:716-21.	Noneligible study design
55	Tian M, Feng D, Chen X, Chen Y, Sun X, Xiang Y, et al. China's rural public health system performance: a cross-sectional study. PLoS One. 2013;8(12):e83822.	Noneligible study design
56	Tian X, Song Y, Zhang X. National Essential Medicines List and policy practice: a case study of China's health care reform. BMC Health Serv Res. 2012;12:401.	Noneligible study design
57	Wang J, Liu X, Wang S, Chen H, Wang X, Zhou W, et al. Short-term differences in drug prices after implementation of the national essential medicines system: A case study in rural Jiangxi Province, China. Indian J Pharmacol. 2015;47(5):535-9.	Noneligible study design
58	Wang L, Wang A, FitzGerald G, Si L, Jiang Q, Ye D. Who benefited from the New Rural Cooperative Medical System in China? A case study on Anhui Province. BMC Health Serv Res. 2016;16:195.	Noneligible study design

59	Wang S, Xu J, Jiang X, Li C, Li H, Song S, et al. Trends in health resource disparities in primary health care institutions in Liaoning Province in Northeast China. Int J Equity Health. 2018;17(1):178.	Noneligible study design
60	Wang X, Tang Y, Liu C, Liu J, Cui Y, Zhang X. Effects of restrictive prescribing on antibiotic consumption in primary care in China, 2012–17: an interrupted time-series analysis. The Lancet. 2019;394.	Noneligible study design
61	Wang Y, Eggleston K, Yu Z, Zhang Q. Contracting with private providers for primary care services: evidence from urban China. Health Econ Rev. 2013;3(1):1.	Noneligible study design
62	Wang Y, Shu Z, Gu J, Sun X, Jing L, Bai J, et al. Evidence for capitation reform in a New Rural Cooperative Medical Scheme in Pudong New Area, Shanghai: A longitudinal study. Int J Health Plann Manage. 2017;32(3):307-16.	Noneligible study design
63	Wang Y, Zhu Y, Shi H, Sun X, Chen N, Li X. The Effect of the Full Coverage of Essential Medicines Policy on Utilization and Accessibility of Primary Healthcare Service for Rural Seniors: A Time Series Study in Qidong, China. Int J Environ Res Public Health. 2019;16(22).	Noneligible study design
64	Wang Z, Chang R, Luo YB, Wu J, Wang Q, Lu ZX, et al. Evaluation of Need and Distribution of National Essential Medicines List in Village Clinics: A Cross-sectional Study Based on the Perspective of Village Doctors in China. Curr Med Sci. 2019;39(4):663-9.	Noneligible study design
65	Wang Z, Chen Y, Pan T, Liu X, Hu H. The comparison of healthcare utilization inequity between URRBMI and NCMS in rural China. Int J Equity Health. 2019;18(1):90.	Noneligible study design
66	Wang Z, Shi J, Wu Z, Xie H, Yu Y, Li P, et al. Changes in chronic disease management among community health centers (CHCs) in China: Has health reform improved CHC ability? Int J Health Plann Manage. 2017;32(3):317-28.	Noneligible study design
67	Wong HT, Guo YQ, Chiu MY, Chen S, Zhao Y. Spatial illustration of health-care workforce accessibility index in China: How far has our 2009 health-care reform brought us? Aust J Rural Health. 2016;24(1):54-60.	Noneligible study design
68	Wu J, Li X, Song Y, Shao H, Shi Q, Qin D, et al. The impact of a bundled policy intervention on improving the performance of rural healthcare in China. Int J Equity Health. 2016;15:46.	Noneligible study design
69	Wu J, Liu J, Zhu B, Mao Y. Does China's new medical reform improve health equity of rural residents? Evidence from household surveys before and after the implementation of new medical reform in Shaanxi Province, China. Value in Health. 2015;18(7):A526.	Noneligible study design
70	Wu L, Pu C. Delivering Contracted Family Doctor Services in Rural Areas during the Implementation of Health Poverty Alleviation Project: Critical Problems and Recommendations. Chinese General Practice. 2019;22(33):4123-7.	Noneligible study design

71	Wu Y, Zhang L, Liu X, Ye T, Wang Y. Geographic variation in health insurance benefits in Qianjiang District, China: a cross-sectional study. Int J Equity Health. 2018;17(1):20.	Noneligible study design
72	Xiang X, Yang C, Wang D, Ye J, Zhang X. Effects of China's national essential medicines policy on the use of injection in primary health facilities. J Huazhong Univ Sci Technolog Med Sci. 2012;32(4):626-9.	Noneligible study design
73	Xiao Y, Wang J, Shen P, Zheng B, Zheng Y, Li L. Retrospective survey of the efficacy of mandatory implementation of the Essential Medicine Policy in the primary healthcare setting in China: failure to promote the rational use of antibiotics in clinics. Int J Antimicrob Agents. 2016;48(4):409-14.	Noneligible study design
74	Xinpu L. The impacts of national essential medicine policies on the rational use of medicines in China: A cross-sectional study in primary health care institution. Journal of Chinese Pharmaceutical Sciences. 2019;28(1):49-55.	Noneligible study design
75	Xu X, Zhou L, Antwi HA, Chen X. Evaluation of health resource utilization efficiency in community health centers of Jiangsu Province, China. Hum Resour Health. 2018;16(1):13.	Noneligible study design
76	Yang C, Shen Q, Cai W, Zhu W, Li Z, Wu L, et al. Impact of the zero-markup drug policy on hospitalisation expenditure in western rural China: an interrupted time series analysis. Trop Med Int Health. 2017;22(2):180-6.	Noneligible study design
77	Yao Q, Liu C, Ferrier JA, Liu Z, Sun J. Urban-rural inequality regarding drug prescriptions in primary care facilities - a pre-post comparison of the National Essential Medicines Scheme of China. Int J Equity Health. 2015;14:58.	Noneligible study design
78	Yin J, Kong AP, Chan JC. Prevention and Care Programs Addressing the Growing Prevalence of Diabetes in China. Curr Diab Rep. 2016;16(12):130.	Noneligible study design
79	Yin J, Li Q, Sun Q. Antibiotic consumption in Shandong Province, China: an analysis of provincial pharmaceutical centralized bidding procurement data at public healthcare institutions, 2012-16. J Antimicrob Chemother. 2018;73(3):814-20.	Noneligible study design
80	Yin S, Song Y, Bian Y. Does the Essential Medicines Policy Succeed in China? Empirical Study on Rational Medicine Use in Primary Health Care Institutions. Ther Innov Regul Sci. 2014;48(6):689-95.	Noneligible study design
81	Yip W, Hsiao W. China's health care reform: A tentative assessment. China Economic Review. 2009;20(4):613-9.	Noneligible study design
82	Yuan L, Shao Y, Ren L, Sun X. Implementation Progress in Performance Appraisal for the Staff of Community Health Institutions in China after New Health Reform. Chinese General Practice. 2018;21(28):3417-22 and 29.	Noneligible study design
83	Zhang D, Shi L, Tian F, Zhang L. Care Utilization with China's New Rural Cooperative Medical Scheme: Updated Evidence from the China Health and Retirement Longitudinal Study 2011-2012. Int J Behav Med. 2016;23(6):655-63.	Noneligible study design

84	Zhang J, Jin RR, Li JJ, Li JL, Su XW, Deng GJ, et al. Study on the effectiveness of implementation: the National Demonstration Areas for Comprehensive Prevention and Control of Non-communicable Diseases. Zhonghua Liu Xing Bing Xue Za Zhi. 2018;39(4):394-400.	Noneligible study design
85	Zhang L, Li S, Yi H, d'Intignano LM, Ding Y. Correlation Between New Cooperative Medical Scheme Policy Design and Catastrophic Medical Payment: Evidence From 25 Counties in Rural China. Asia Pac J Public Health. 2016;28(1):26-38.	Noneligible study design
86	Zhang YG, Dong D, Xu L, Miao ZW, Mao WH, Tang SL. Equity in health care after 10 years of the New Rural Co-operative Medical Insurance Scheme in China: an analysis of national survey data. Lancet. 2018;392(Supplement 1):35-.	Noneligible study design
87	Zhao P, Diao Y, You L, Wu S, Yang L, Liu Y. The influence of basic public health service project on maternal health services: an interrupted time series study. BMC Public Health. 2019;19(1):824.	Noneligible study design
88	Zhao P, Han X, You L, Zhao Y, Yang L, Liu Y. Effect of basic public health service project on neonatal health services and neonatal mortality in China: a longitudinal time-series study. BMJ Open. 2020;10(7):e034427.	Noneligible study design
89	Zhou L, Xu X, Antwi HA, Wang L. Towards an equitable healthcare in China: evaluating the productive efficiency of community health centers in Jiangsu Province. Int J Equity Health. 2017;16(1):89.	Noneligible study design
90	Zhou S, Xu J, Ma X, Yuan B, Liu X, Fang H, et al. How Can One Strengthen a Tiered Healthcare System through Health System Reform? Lessons Learnt from Beijing, China. Int J Environ Res Public Health. 2020;17(21).	Noneligible study design
91	Zhou Y, Qin JM, Tang JX, Chen Y, Zhang LF, Zhang YC. Benefit Incidence and Equity of Government Medical Subsidy Received by People Under Different Health Insurance. Chinese General Practice. 2016;19(22):2710-3.	Noneligible study design
92	Zhou YJ, Tang YX, Zhang Q, Qiu PY, Zhang Q. Catastrophic Health Expenditure before and after Compensations from the New Rural Cooperative Medical Scheme in Rural Zigong of Sichuan Province. Sichuan Da Xue Xue Bao Yi Xue Ban. 2016;47(5):768-71.	Noneligible study design
93	Chao J, Gu J, Zhang H, Chen H, Wu Z. The Impact of the National Essential Medicines Policy on Rational Drug Use in Primary Care Institutions in Jiangsu Province of China. Iranian journal of public health. 2018 Jan 1;47(1):24-32.	Noneligible study design
94	Cheng JM, Yuan YX, Lu W, Yang L. Primary health care in China: is China's health reform reform for the whole nation? Prim Health Care Res Dev. 2017;18(4):398-403.	Noneligible study design
95	Dai T, Hu HP, Na X, Li YZ, Wan YL, Xie LQ. Effects of New Rural Cooperative Medical Scheme on Medical Service Utilization and Medical Expense Control of Inpatients: A 3-year Empirical Study of Hainan Province in China. Chin Med J (Engl). 2016;129(11):1280-4.	Noneligible study design

96	Ding H, Chen Y, Yu M, Zhong J, Hu R, Chen X, Wang C, Xie K, Eggleston K. The effects of chronic disease management in primary health care: Evidence from rural China. Journal of Health Economics. 2021 Dec 1;80:102539.	Noneligible study design
97	Dong CH, Fan CS, Zheng J. Medical Expenditure for People with Diabetes in Urban Employee Basic Medical Insurance Program in Beijing. Value in Health. 2013;16(3):A161-A2.	Noneligible study design
98	Fan X, Su M, Si Y, Zhao Y, Zhou Z. The benefits of an integrated social medical insurance for health services utilization in rural China: evidence from the China health and retirement longitudinal study. International journal for equity in health. 2021 Dec;20(1):1-0.	Noneligible study design
99	Feng D, Zhang D, Li B, Zhang Y, Serrano R, Shi D, et al. Does having a usual primary care provider reduce patient self-referrals in rural China's rural multi-tiered medical system? A retrospective study in Qianjiang District, China. BMC Health Serv Res. 2017;17(1):778.	Noneligible study design
100	Feng X, Feng W, Shen P, Wang Z, Shen J, Wang B. The effect of the integrated delivery system on managing hypertension in rural areas of China. Annals of Palliative Medicine. 2021 Jan 1;10(1):434-42.	Noneligible study design
101	Feng XL, Pang M, Beard J. Health system strengthening and hypertension awareness, treatment and control: data from the China Health and Retirement Longitudinal Study. Bull World Health Organ. 2014;92(1):29-41.	Noneligible study design
102	Gong Y, Xu J, Chen T, Sun N, Lu Z, Yin X. The effect of the latest health care reforms on the quality of community health services in China. Int J Health Plann Manage. 2018;33(4):e1225-e31.	Noneligible study design
103	Green C, Hollingsworth B, Yang M. The impact of social health insurance on rural populations. The European Journal of Health Economics. 2021 Apr;22(3):473-83.	Noneligible study design
104	Huang J, Liu Y, Zhang T, Wang L, Liu S, Liang H, Zhang Y, Chen G, Liu C. Can family doctor contracted services facilitate orderly visits in the referral system? A frontier policy study from Shanghai, China. The International Journal of Health Planning and Management. 2022 Jan;37(1):403-16.	Noneligible study design
105	Liu Q, Tian X, Tian J, Zhang X. Evaluation of the effects of comprehensive reform on primary healthcare institutions in Anhui Province. BMC Health Services Research. 2014 Dec;14(1):1-7.	Noneligible study design
106	Liu S, Lin J, He Y, Xu J. The Service Capability of Primary Health Institutions under the Hierarchical Medical System. InHealthcare 2022 Feb 10 (Vol. 10, No. 2, p. 335). MDPI.	Noneligible study design
107	Nie L. Analysis of the New Rural Cooperative Medical System and Its Countermeasures. In 2017 International Conference on Management Science and Management Innovation (MSMI 2017) 2017 Jun (pp. 250-251). Atlantis Press.	Noneligible study design

108	Qin C, Liu M, Guo X, Liu J. Human Resources in Primary Health-Care Institutions before and after the New Health-Care Reform in China from 2003 to 2019: An Interrupted Time Series Analysis. International Journal of Environmental Research and Public Health. 2022 May 16;19(10):6042.	Noneligible study design
109	Tang Y, Zhang X, Yang C, Yang L, Wang H, Zhang X. Application of propensity scores to estimate the association between government subsidy and injection use in primary health care institutions in China. BMC health services research. 2013 Dec;13(1):1-7.	Noneligible study design
110	Xu W, Cao N, Li X, Jiang J. The effect of basic medical insurance on the changes of primary care seeking behavior: An application of hierarchical age-period-cohort analysis. Frontiers in public health. 2022 Aug 3:2410.	Noneligible study design
111	Yan C, Liao H, Ma Y, Wang J. The Impact of Health Care Reform Since 2009 on the Efficiency of Primary Health Services: A Provincial Panel Data Study in China. Frontiers in Public Health. 2021;9.	Noneligible study design
112	Yang L, Sun L, Wen L, Zhang H, Li C, Hanson K, Fang H. Financing strategies to improve essential public health equalization and its effects in China. International journal for equity in health. 2016 Dec;15(1):1-2.	Noneligible study design
113	Zhang L, Li J, Ma T, Zhang R, Zhang Q. Usual source of care and experiences with primary care among community health service centre patients in Changchun, China: a cross-sectional survey. Health & Social Care in the Community. 2020 Nov;28(6):1979-88.	Noneligible study design
114	Zhou W, Li X, Qian Q. Comparison of Gatekeeping and Non-gatekeeping Designs in a Service System with Delay-sensitive Customers. Journal of Systems Science and Systems Engineering. 2021 Apr;30(2):125-50.	Noneligible study design
115	丁骏, 沈宇清, 仇爱红, 李少冬. 基本药物制度实施对江苏省中部某县级市乡镇卫生院运行绩效的影响. 医学与社会. 2014;27(12):35-7.	Noneligible study design
116	严周忠, 黄晓光. 基本药物制度对江苏省某市乡镇卫生院经济运行的影响. 医学与社会. 2014;27(8):42-5.	Noneligible study design
117	刘军安, 罗庆, 刘欢, 梁渊, 孙奕, 卢祖洵. 国家基本药物制度下的村卫生室处方费用及影响因素分析. 中国卫生经济. 2014;12.	Noneligible study design
118	刘向容. 总额预付和增加基层医疗资源对分级诊疗的影响——基于 CHARLS 数据的实证分析. 中国卫生政策研究. 2016;9(4):16-22.	Noneligible study design

119	刘智勇, 籍文雪, 姚强, 罗飞, 何露洋, 陈山泉, et al. 基本药物制度对基层医疗机构收支结构的影响分析: 基于上海, 四川与重庆地区实证研究. 中国卫生经济. 2014;33(1):21-3.	Noneligible study design
120	刘艳, 朱福, 张蓉蓉, 毛士龙. 实施国家基本药物制度对上海市某社区卫生服务中心门诊用药的影响. 中国药房. 2016;27(18):2476-9.	Noneligible study design
121	刘黎明, 程薇, 蒋艳, 赵丽颖, 徐阅, 娄翠迪, et al. 北京市医药分开综合改革后慢性病人群治疗费用的机构流向分析——基于 “SHA2011”. 中国卫生政策研究. 2021;14(4):11-7.	Noneligible study design
122	史航, 王莹, 刘平羽, 孙新华, 李歆. 启东市农村老年患者基层医疗机构基本药物全额保障政策的效果评价. 南京医科大学学报: 社会科学版. 2020;20(3):215-9.	Noneligible study design
123	吴敏, 刘岩, 喻倩, 白同禹, 王爱文. 山东省基层医疗卫生机构综合改革的政策效应. 卫生经济研究. 2012;12.	Noneligible study design
124	吴泽兵, 王前强, 朱平华, 杨森. 广西乡镇卫生院实施基本药物制度前后经济运行情况比较. 中国卫生经济. 2013;32(12):81-4.	Noneligible study design
125	周英达, 卓书雄. 医联体全科团队建设下的居民家庭医生签约意愿分析. 山西医药杂志. 2020.	Noneligible study design
126	周蓉. 基本药物制度对基层医疗卫生机构合理用药的影响. 世界最新医学信息文摘 (电子版). 2017(92):136-.	Noneligible study design
127	周风玲. 医改后的社区卫生服务中心经济运行分析——以南京市江宁区为例. 经济师. 2020;4.	Noneligible study design
128	孙义芳. 基本药物制度对基层医疗卫生机构抗生素使用的影响. 医疗装备. 2016;29(10):126-7.	Noneligible study design
129	孙华君, 田慧, 杜沩. 家庭医生签约服务对居民就诊行为的影响: 基于倾向得分匹配的实证研究. 中国全科医学. 2020;23(19):2396.	Noneligible study design

130	孙葵, 尹文强, 黄冬梅, 于倩倩, 赵延奎, 李云伟, et al. 新医改前后基层医疗机构医生队伍稳定性变化趋势及影响因素研究. 中国卫生事业管理. 2016;33(12):912-4.	Noneligible study design
131	宋熠春, 李苏平, 周守君, 胡晓抒. 基本药物制度对基层医疗机构运行的影响. 江苏医药. 2011;37(17):2091-2.	Noneligible study design
132	宋燕, 卞鹰. 基本药物制度实施后基层医疗卫生机构药品价格变动分析——基于中国四省 (自治区) 的实证研究. 卫生经济研究. 2013(4):48-51.	Noneligible study design
133	宋燕, 卞鹰. 实施国家基本药物制度对山东省某县乡镇卫生院的影响调研及政策建议. 中国药房. 2013;24(8):693-5.	Noneligible study design
134	宫志强, 万泉, 王从从, 郭锋, 赵郁馨. 基本药物制度实施对六省乡镇卫生院经济运行影响分析. 中国卫生经济. 2014;33(6):65-8.	Noneligible study design
135	宫怡飞. 探讨国家基本药物制度对基层医疗机构合理用药影响. 中西医结合心血管病杂志 (电子版). 2017;5(33):163-4.	Noneligible study design
136	张利, 宋沈超. 新医改前后贵州省乡镇卫生院收支结构变化. 贵阳医学院学报. 2015;40(1):28-31.	Noneligible study design
137	张彤, 黄迎春. 社区卫生服务机构收支两条线管理实施效果分析. 中国卫生经济. 2009;28(7):38-41.	Noneligible study design
138	张燕燕, 王锦帆. 江苏省基层医疗卫生机构改革成效研究. 中国全科医学. 2020;23(1):25.	Noneligible study design
139	张玲玲, 孙华君, 梁黎明, 陈欣, 杜汋. 签约家庭医生对天津市居民医联体认知的影响. 医学与社会. 2020;33(6):26-9.	Noneligible study design
140	张邹, 孙静, 张笑天. 社区首诊制下定点基层医疗机构门诊服务利用水平分析. 电子测试. 2014(10):118-9.	Noneligible study design
141	景日泽, 来晓真. 基于两水平方差成分模型的家庭医生签约服务费对家庭医生诊疗行为和患者医疗费用的影响研究. 中国全科医学. 2021;24(4):392.	Noneligible study design

142	李叶菡, 初炜, 车成新, 李云, 王晶晶. 实施药品零差率销售对基层医疗机构收入的影响与补偿研究. 中国卫生事业管理. 2014;31(3):191-3.	Noneligible study design
143	李宝玉, 刘国祥, 张清华, 马祎. 基本药物零差率政策的实施对乡镇卫生院经营管理的影响研究 2012.	Noneligible study design
144	李新泰, 王文华, 尹爱田. 山东省基本药物制度对乡镇卫生院合理用药的影响. 中国卫生经济. 2011;30(4):22-3.	Noneligible study design
145	杜娟, 郑淑美, 徐俊杰, 赵亚利, 王慧丽, 郭爱民, et al. 北京市社区卫生服务机构基本药物 “零差率” 政策对农村高血压患者治疗费用的影响. 中国全科医学. 2013;16(7):737-40.	Noneligible study design
146	杨俐. 基本药物制度实施前后长风社区卫生服务中心口服降糖药用药分析. 上海医药. 2015;36(6):19-22.	Noneligible study design
147	杨文燕, 王文华, 尹爱田. 医改前后山东省乡镇卫生院资源配置与利用情况比较分析. 中国卫生经济. 2011;30(12):28-30.	Noneligible study design
148	杨非衡, 倪娜娜, 朱磊, 杨桦, 高运生, 何欢, et al. 北京市朝阳区社区卫生服务机构绩效考核改革效果研究. 中国全科医学. 2016;19(04):386.	Noneligible study design
149	武宁, 杨洪伟. 基本药物制度对基层医疗卫生机构合理用药的影响. 卫生经济研究. 2013(9):47-8.	Noneligible study design
150	汤真清, 何江江, 唐密, 张天晔, 钟姮, 李莉丽, et al. 上海市药物使用联动机制对医疗机构门诊服务利用结构的影响: 基于中断时间序列模型. 中国全科医学. 2019;22(28):3415.	Noneligible study design
151	王世建, 徐文. 探讨基本药物制度实行对陕西省基层医疗机构合理用药的影响. 西部中医药. 2017;30(3):89-92.	Noneligible study design
152	王志翊, 王仲, 翁杰, 侯若南, 吴和, 胡公义, et al. 医联体全科团队对居民家庭医生签约意愿的影响研究. 中国全科医学. 2018;21(27):3389.	Noneligible study design
153	王明芳. 乡镇卫生院实施国家基本药物制度影响分析. 中国农村卫生事业管理. 2013(3):250-2.	Noneligible study design

154	王洪军, 吴爱华. 基层医疗机构实行药品零差价后对居民医疗费用变化的影响因素分析. 中国卫生经济. 2011;30(4):68-9.	Noneligible study design
155	王清波, 杨洪伟, 赵锋, 田磊磊, 杨莉. 基本药物制度实施后村卫生室门诊服务变化比较——基于东, 中, 西部 6 省的抽样调查. 中国卫生政策研究. 2013;6(12):33-40.	Noneligible study design
156	王芳. 基本药物制度对社区基层医疗机构良性运行的影响. 当代临床医刊. 2016;29(1):1871-2.	Noneligible study design
157	王进雄, 谭林, 胡君. 实施社区首诊制对社区卫生服务机构的质量控制影响因素分析. 齐齐哈尔医学院学报. 2016;37(32):4069-70.	Noneligible study design
158	皮星, 罗长坤, 康军, 陈黎明, 陈自强, 吴玉林. 乡镇卫生院 “药品零加成” 成效分析和建议: 以重庆市江北区乡镇卫生院改革为例. 中国卫生质量管理. 2010;17(5):98-100.	Noneligible study design
159	盛红旗, 蔡伟芹, 王黎勇, 陈秀芝, 王宪祥, 马安宁. 基本药物制度实施前后乡镇卫生院效率变化研究. 中国卫生经济. 2014;33(11):38-41.	Noneligible study design
160	祁鸽, 刘文轩, 李亦兵. 新医改前后我国基层医疗卫生机构服务能力研究. 中国药物经济学. 2022.	Noneligible study design
161	祝新. 探讨国家基本药物制度对基层医疗机构合理用药影响. 中国卫生产业. 2018;15.	Noneligible study design
162	秦江梅, 明延飞, 林春梅, 毛璐, 张艳春, 张丽芳. 新医改以来我国基层医疗卫生机构的效率变动分析. 中国卫生经济. 2015(8):21-3.	Noneligible study design
163	罗力, 李婉莹, 李强, 邵月琴, 沈宏, 陈海乐, et al. 上海市实施国家基本药物制度对社区卫生服务中心合理用药的影响. 中国药房. 2013;24(4):289-91.	Noneligible study design
164	罗艳, 冯辉, 何国平, 罗昶, 付兆亮, 尹春春, et al. 城乡居民医保社区门诊统筹对提高基层医疗服务利用的效果研究. 中国全科医学. 2013;16(4):377-9.	Noneligible study design

165	罗飞, 姚岚, 姚强, 何露洋, 刘红玉, 潘瑶, et al. 国家基本药物制度对中西部基层医疗机构合理用药的影响. 中国医院管理. 2013(6):41-3.	Noneligible study design
166	薛文浩, 莫旦红, 钟华, 张彩丽, 戴丹华. 社区综改数据分析系统对家庭医生签约后有效服务的效果评价. 中国社区医师. 2020.	Noneligible study design
167	袁磊, 赵志刚, 王亚魁, 陈仲强, 韩庆峰, 李颜. 北京市双向转诊制度实施情况及居民对基层医疗的满意度调查. 中国全科医学. 2017;20(z2):216-9.	Noneligible study design
168	袁莎莎, 贾梦, 王芳, 赵君, 李熹, 谭雯, et al. 不同医联体模式下基层医疗机构与上级医院协作机制比较分析. 中国卫生事业管理. 2019;36(2):81-3.	Noneligible study design
169	覃娴静, 徐婷婷, 高洪达, 陈奕如, 江南, 曾丽蓉, et al. 县域医共体改革后乡镇卫生院医疗服务能力的变化. 广西医学. 2018;40(24):2932-5.	Noneligible study design
170	贺小林, 梁鸿. 社区卫生服务门诊统筹政策的成效与经验 2011.	Noneligible study design
171	贾梦, 王芳, 田淼淼, 袁莎莎, 赵敏捷. 县域医共体试点对基层医疗卫生机构的早期影响研究. 中国农村卫生事业管理. 2020;40(7):482-5.	Noneligible study design
172	赵临, 杨立成. 分级诊疗实施前后天津市社区卫生服务中心运行效率评价研究. 中国全科医学. 2020;23(1):30.	Noneligible study design
173	赵文聪, 武宁, 赵秀竹. 基本药物制度对江西五所基层医疗卫生机构合理用药的影响. 中国卫生经济. 2012;31(12):60-1.	Noneligible study design
174	赵锋, 杨洪伟, 林郅中, 左延莉, 田磊磊, 杨莉. 南宁市基本药物制度实施前后十七家乡镇卫生院卫生服务变化分析. 中国卫生经济. 2013(1):28-30.	Noneligible study design
175	赵锋, 杨洪伟, 林郅中, 左延莉, 田磊磊, 杨莉. 基本药物制度实施后广西某市社区卫生服务中心与乡镇卫生院门诊服务变化比较. 中国卫生政策研究. 2012;5(11):19-26.	Noneligible study design
176	辛喜萍. 国家基本药物制度实施对社区卫生机构的影响. 中国社区医师: 医学专业. 2012;14(31):32-.	Noneligible study design

177	连妍超, 闫娟娟, 郭跃铭. 基本药物制度对山西省某市乡镇卫生院经济运行的影响. 中国卫生经济. 2016;35(8):88-90.	Noneligible study design
178	连颖菁, 李跃平. 医联体内基层医疗机构运行效率的影响研究. 现代医院管理. 2019;17(3):16-9.	Noneligible study design
179	邹榕, 罗红叶, 黎燕宁, 胡振, 冯启明. 国家基本药物制度对广西乡镇卫生院门诊用药的影响研究. 中国全科医学. 2012;15(13):1451-3.	Noneligible study design
180	邹鑫, 韩亚蓉, 管神艺, 唐泽亀, 邱敏, 张树琴, et al. 家庭医生激励对 2 型糖尿病患者医疗费用的影响. 中国全科医学. 2021;24(13):1644.	Noneligible study design
181	郑思茜, 宋燕, 殷实, 卞鹰. 基本药物制度对宁夏基层医疗卫生机构门诊处方合理用药的影响. 中国初级卫生保健. 2014;28(8):1-2.	Noneligible study design
182	郭洪伟, 尹文强, 赵延奎, 黄冬梅, 胡金伟, 于倩倩. 基本药物制度实施后 J 市乡镇卫生院医疗人力资源变化趋势研究. 中国卫生事业管理. 2014;31(7):507-9.	Noneligible study design
183	郭胜, 李江峰, 唐立岷. 分级诊疗制度下我国基层医疗卫生机构发展现状分析. 中国初级卫生保健. 2019;33(10):32-4.	Noneligible study design
184	陈丽, 杨洪伟, 梁小云, 李珍, 李佳, 金承刚. 基本药物制度对基层卫生机构月门诊量的影响评价. 中国医院管理. 2013(11):72-3.	Noneligible study design
185	陈斌, 陈晓燕, 陆海峰, 栾伟, 李挺. 基于提高签约居民门诊就诊对应率的家庭医生服务改革成效研究. 中国全科医学. 2020;23(4):463.	Noneligible study design
186	陈昆, 黄玉萍, 陈莹, 廖小兵. 国家基本药物制度实施对社区卫生服务机构的影响研究. 北方药学. 2015;12(9):196-.	Noneligible study design
187	陈洁, 彭江丽, 赵朋娟, 刘晖, 王璐, 张丽萍. 基本药物制度对昆明市基层医疗机构门诊合理用药的影响. 昆明医科大学学报. 2014(3):83-7.	Noneligible study design
188	陈瑶, 白冰, 代涛. 安徽省基层医疗卫生机构基本药物制度实施效果. 中国卫生政策研究. 2013(4):31-5.	Noneligible study design

189	陈秀芝, 马安宁, 蔡伟芹, 张玉. 基本药物制度对乡镇卫生院的影响探究. 中国初级卫生保健. 2013;27(7):24-5.	Noneligible study design
190	陈蕾, 冷明祥, 唐晓东, 胡大洋, 万彬, 程向前. 不同医保支付方式下社区卫生服务机构运行情况分析. 医学与哲学: A. 2012;33(6):60-2.	Noneligible study design
191	雷坤, 赵炜, 贾利高, 王全. 医疗联合体对社区卫生服务中心运行效益的影响. 数理医药学杂志. 2014;27(1):108-9.	Noneligible study design
192	韩娟, 张雪梅, 赵志忠, 马骉, 郝继影, 崔远利, et al. 北京市昌平区社区卫生服务中心基本药物制度实施效果的评价. 首都食品与医药. 2015(16):13-5.	Noneligible study design
193	章小敏, 陈翔, 陈将, 鲍俞燕, 叶爱菊, 洪冰. 分级诊疗制度下不同级别医院间糖尿病基本药物使用现状研究. 中国全科医学. 2021;24(12):1546.	Noneligible study design
194	高力军, 李叶, 李熹, 吴群红. 国家基本药物制度下黑龙江省 “卫十一项目” 地区乡镇卫生院用药合理性评价. 中国药房. 2015;26(27):3745-7.	Noneligible study design
195	魏艳, 尹文强, 马欣, 黄冬梅, 于倩倩, 范海平, et al. 基本药物制度对山东省乡镇卫生院药品可负担性影响研究. 中国卫生经济. 2013;32(10):18-20.	Noneligible study design
196	黄普明, 陈伟薇, 吕雄文, 解雪峰, 黄少玉, 叶婷婷, et al. 新医改背景下肥西县乡镇卫生院用药合理性的调查. 安徽医药. 2012;16(2):256-8.	Noneligible study design
197	黄杰, 杨洪伟, 杨莉, 樊海涛, 金承刚, 汪雪莲, et al. 陕西省基本药物制度对基层卫生机构的影响——基于两市 (县) 4 所基层医疗机构的抽样调查. 中国卫生政策研究. 2011;4(11):1-6.	Noneligible study design
198	黄蛟灵, 方帅, 梁鸿, 刘姗姗, 王良晨, 芦炜, et al. 家庭医生签约服务协同改革对居民健康管理的影响. 中国卫生资源. 2018;21(4):329-32.	Noneligible study design
199	孙俊伟, 程永福, 李娟, 张艳. 宁波市江北区社区卫生服务综合配套改革的政策评价. 中国卫生政策研究. 2010;3(7):26-31.	Noneligible study design

200	李熹, 吴群红, 高力军, 郝模. 国家基本药物制度实施对某省乡镇卫生院卫生服务行为及运营状况的影响评价. 中国药房. 2015;26(30):4177-81.	Noneligible study design
201	杜健, 张金钟, 杨宝贵, 周航. 国家基本药物制度对社区卫生服务机构发展及患者满意度影响的调查分析. 中国药房. 2014;25(16):1450-2.	Noneligible study design
202	蔡木禹, 林朝仙, 李艳萍, 罗小院, 李展星. 实施国家基本药物制度前后汕头市潮南区基层医疗卫生机构临床用药情况调查. 中国药房. 2013;24(28):2605-7.	Noneligible study design
203	谢冬玲, 马娇, 崔浩, 岳琳, 韩晖. 我国东, 中, 西部部分县 (区) 村卫生室国家基本药物制度实施前后处方分析. 中国药房. 2014;25(48):4523-5.	Noneligible study design
204	邓伟中, 蔡胜娣, 吴丽萍, 贺惠娴. 家庭医生服务式健康教育对社区 2 型糖尿病患者治疗效果的影响. 海南医学. 2016(12):2039-40.	Noneligible study design
205	Song Y, Bian Y. Does National Essential Medicine System Improve Rational Drug Use in Primary Health Care Facilities? An Empirical Study in Rural China. Value in Health. 2012 Nov 1;15(7):A613.	Noneligible study design
206	Yip W, Powell-Jackson T, Chen W, Hu M, Fe E, Hu M, Jian W, Lu M, Han W, Hsiao WC. Capitation combined with pay-for-performance improves antibiotic prescribing practices in rural China. Health affairs. 2014 Mar 1;33(3):502-10.	Noneligible study design
207	Jing L, Liu K, Zhou X, Wang L, Lou J, Sun X. Effectiveness of an incentive policy intervention for rural health-care providers: a longitudinal survey in Shanghai. The Lancet. 2016;388.	Not PHC outcomes
208	Li C, Tang C, Wang H. Effects of health insurance integration on health care utilization and its equity among the mid-aged and elderly: evidence from China. Int J Equity Health. 2019;18(1):166.	Not PHC outcomes
209	Liu K, Jing LM, Zhou XH, Huang Y, Wang L, Sun XM. The health brain-drain in rural areas about the health personnel incentive policy intervention in Shanghai: a longitudinal study. Lancet. 2016;388:44-.	Not PHC outcomes
210	Mo L, Yang X, He J, Dong B. Evaluation of potentially inappropriate medications in older inpatients in China. J Am Geriatr Soc. 2014;62(11):2216-8.	Not PHC outcomes
211	Su D, Chen YC, Gao HX, Li HM, Chang JJ, Jiang D, et al. Effect of integrated urban and rural residents medical insurance on the utilisation of medical services by residents in China: a propensity score matching with difference-in-differences regression approach. BMJ Open. 2019;9(2):e026408.	Not PHC outcomes

212	Tan SY, Wu X, Yang W. Impacts of the type of social health insurance on health service utilisation and expenditures: implications for a unified system in China. Health Econ Policy Law. 2019;14(4):468-86.	Not PHC outcomes
213	Wan X, Lu Q, Zhu T. Application effect of integration and sharing model in progressive diabetes comprehensive training of community nurse in Wuxi. Chinese Nursing Research. 2018;32(16):2580-3.	Not PHC outcomes
214	Zhou Y, Rosenheck RA, He H. Health insurance in China: variation in co-payments and psychiatric hospital utilization. J Ment Health Policy Econ. 2014;17(1):25-32.	Not PHC outcomes
215	Chen J, Xu S, Gao J. The Mixed Effect of China's New Health Care Reform on Health Insurance Coverage and the Efficiency of Health Service Utilisation: A Longitudinal Approach. Int J Environ Res Public Health. 2020;17(5).	Not PHC outcomes
216	Chung VC, Ma PH, Wang HH, Wang JJ, Hong LC, Wei X, et al. Integrating traditional chinese medicine services in community health centers: insights into utilization patterns in the pearl river region of china. Evid Based Complement Alternat Med. 2013;2013:426360.	Not PHC outcomes
217	Dong H, Duan S, Bogg L, Wu Y, You H, Chen J, et al. The impact of expanded health system reform on governmental contributions and individual copayments in the new Chinese rural cooperative medical system. Int J Health Plann Manage. 2016;31(1):36-48.	Not PHC outcomes
218	Feng Y, Xiong X, Xue Q, Yao L, Luo F, Xiang L. The impact of medical insurance policies on the hospitalization services utilization of people with schizophrenia: A case study in Changsha, China. Pak J Med Sci. 2013;29(3):793-8.	Not PHC outcomes
219	Fu H, Li L, Yip W. Intended and unintended impacts of price changes for drugs and medical services: Evidence from China. Soc Sci Med. 2018;211:114-22.	Not PHC outcomes
220	He W. Effects of establishing a financing scheme for outpatient care on inpatient services: empirical evidence from a quasi-experiment in China. The European Journal of Health Economics. 2022 Feb;23(1):7-22.	Not PHC outcomes
221	Jing LM, Liu K, Zhou XH, Sun XM, Wang L, Huang Y, et al. Effectiveness Evaluation of the Reform in Incentive System for Healthcare Professionals Implemented. Chinese General Practice. 2017;20(19):2329-33.	Not PHC outcomes
222	Ren Y, Zhou Z, Cao D, Ma BH, Shen C, Lai S, Chen G. Did the Integrated Urban and Rural Resident Basic Medical Insurance Improve Benefit Equity in China?. Value in Health. 2022 May 3.	Not PHC outcomes
223	Zhaokang Y, Yuxi L, Yong L, Yunchang X, Yuanjun G, Harris M. A model for community health service development in depressed rural areas in China. BMC Health Services Research. 2012 Dec;12(1):1-6.	Not PHC outcomes

224	吴妮娜, 周海清, 刘慧, 常文虎. 社区卫生服务中心不同收支两条线管理模式下的服务效率分析——基于北京市远郊区县的实证研究. 中国社会医学杂志. 2012(2):131-3.	Not PHC outcomes
225	朱凤梅. 医保预付制改革的效果研究——来自中国 CHIRA 数据的实证检验. 保险研究. 2021(3):112-27.	Not PHC outcomes
226	Zhu D, Shi X, Nicholas S, Bai Q, He P. Impact of China's healthcare price reforms on traditional Chinese medicine public hospitals in Beijing: an interrupted time-series study. BMJ Open. 2019;9(8):e029646.	Not PHC outcomes
227	He Y, Dou G, Huang Q, Zhang X, Ye Y, Qian M, et al. Does the leading pharmaceutical reform in China really solve the issue of overly expensive healthcare services? Evidence from an empirical study. PLoS One. 2018;13(1):e0190320.	Not PHC reform
228	Hesketh T, Wang W, Xu Y, Zhou X. Effect of an incentive-led intervention to address the shortage of village health workers in China: A communitybased, non-randomised controlled trial. The Lancet. 2017;390(SPEC.ISS 1):5.	Not PHC reform
229	Hou Z, Van de Poel E, Van Doorslaer E, Yu B, Meng Q. Effects of NCMS on access to care and financial protection in China. Health Econ. 2014;23(8):917-34.	Not PHC reform
230	Jin Y, Yuan B, Zhu W, Zhang Y, Xu L, Meng Q. The interaction effect of health insurance reimbursement and health workforce on health care-seeking behaviour in China. Int J Health Plann Manage. 2019;34(3):900-11.	Not PHC reform
231	Kong JX, Zhu L, Wang HM, Li Y, Guo AY, Gao C, et al. Effectiveness of the Chronic Care Model in Type 2 Diabetes Management in a Community Health Service Center in China: A Group Randomized Experimental Study. J Diabetes Res. 2019;2019:6516581.	Not PHC reform
232	Lee C, Sun H, Guan Q, Wasserman M. The Effect of China's Basic Medical Insurance Schemes on Health Service Utilization. Value Health. 2014;17(7):A428.	Not PHC reform
233	Lee S, Wang W, Washburn DJ, Shi H, Yu Y, Du Y, et al. Effect of the treatment-before-deposit policy on trust in physicians and perceived service quality among patients in 12 hospitals in China. Int J Health Plann Manage. 2018;33(4):1110-20.	Not PHC reform
234	Lei X, Lin W. The New Cooperative Medical Scheme in rural China: does more coverage mean more service and better health? Health Econ. 2009;18 Suppl 2:S25-46.	Not PHC reform
235	Li YJ, Zou ZH, Yi SY, Yu CY. Effects of Community Management Combined with Self-management on Treatment Compliance and Quality of Life in Elderly Patients with Type 2 Diabetes. Chinese General Practice. 2018;21(26):3217-22.	Not PHC reform

236	Liang J, Zheng X, Chen Z, Dai S, Xu J, Ye H, et al. The experience and challenges of healthcare-reform-driven medical consortia and Regional Health Information Technologies in China: A longitudinal study. Int J Med Inform. 2019;131:103954.	Not PHC reform
237	Liu J, Qiao Y, Gan Y, Lu Z. Evaluation of Family Doctor Services and Intention of Renewing the Contract with the Family Doctor among Contracted Residents in Shenzhen. Chinese General Practice. 2020;23(1):40-4 and 50.	Not PHC reform
238	Mao W, Zhang L, Hu M, Chen W. Evaluation of the enrolment of health insurance after the integration between the New Cooperative Medical Scheme and the Urban Residents Basic Medical Insurance scheme in Hangzhou, China: a longitudinal study. The Lancet. 2018;392.	Not PHC reform
239	Tang Y, Liu C, Zhang X. Public reporting as a prescriptions quality improvement measure in primary care settings in China: variations in effects associated with diagnoses. Sci Rep. 2016;6:39361.	Not PHC reform
240	Tourlomousis F, Chang RC. Dimensional Metrology of Cell-matrix Interactions in 3D Microscale Fibrous Substrates. Procedia CIRP. 2017;65:32-7.	Not PHC reform
241	Wang X, Tang Y, Liu C, Liu J, Cui Y, Zhang X. Effects of restrictive-prescribing stewardship on antibiotic consumption in primary care in China: an interrupted time series analysis, 2012-2017. Antimicrob Resist Infect Control. 2020;9(1):159.	Not PHC reform
242	Wei X, Yin J, Walley JD, Zhang Z, Hicks JP, Zhou Y, et al. Impact of China's essential medicines scheme and zero-mark-up policy on antibiotic prescriptions in county hospitals: a mixed methods study. Trop Med Int Health. 2017;22(9):1166-74.	Not PHC reform
243	Wong CKH, Fung CSC, Yu EYT, Wan EYF, Chan AKC, Lam CLK. Temporal trends in quality of primary care for patients with type 2 diabetes mellitus: A population-based retrospective cohort study after implementation of a quality improvement initiative. Diabetes Metab Res Rev. 2018;34(2).	Not PHC reform
244	Xiang L, Pan Y, Hou S, Zhang H, Sato KD, Li Q, et al. The impact of the new cooperative medical scheme on financial burden of tuberculosis patients: evidence from six counties in China. Infect Dis Poverty. 2016;5:8.	Not PHC reform
245	Xie X, Jin X, Zhang L, Sun H, Shen A, Huang X, et al. Trends analysis for drug utilization in county public hospitals: a sample study of the pilot area of health care reform in China. BMC Health Serv Res. 2018;18(1):812.	Not PHC reform
246	Xiong X, Zhang Z, Ren J, Zhang J, Pan X, Zhang L, et al. Impact of universal medical insurance system on the accessibility of medical service supply and affordability of patients in China. PLoS One. 2018;13(3):e0193273.	Not PHC reform
247	Xu W, Pan Z, Lu S, Zhang L. Regional Heterogeneity of Application and Effect of Telemedicine in the Primary Care Centres in Rural China. Int J Environ Res Public Health. 2020;17(12).	Not PHC reform

248	Yin Z, Perry J, Duan X, He M, Johnson R, Feng Y, et al. Cultural adaptation of an evidence-based lifestyle intervention for diabetes prevention in Chinese women at risk for diabetes: results of a randomized trial. Int Health. 2018;10(5):391-400.	Not PHC reform
249	You H, Gu H, Ning W, Zhou H, Dong H. Comparing Maternal Services Utilization and Expense Reimbursement before and after the Adjustment of the New Rural Cooperative Medical Scheme Policy in Rural China. PLoS One. 2016;11(7):e0158473.	Not PHC reform
250	Yuan B, Li J, Wu L, Wang Z. Multi-Level Social Health Insurance System in the Age of Frequent Employment Change: The Urban Unemployment-Induced Insurance Transition and Healthcare Utilization in China. Healthcare (Basel). 2019;7(2).	Not PHC reform
251	Yuan GL, Qin JM, Liu J, Lin CM, Zhang LF, Zhang YC. Hypertension Control Status and Influencing Factors in Representative Areas of Western China during the Comprehensive Reform of Primary Care System. Chinese General Practice. 2018;21(22):2724-8.	Not PHC reform
252	Zang X, Zhang M, Wei S, Tang W, Jiang S. Impact of public hospital pricing reform on medical expenditure structure in Jiangsu, China: a synthetic control analysis. BMC Health Serv Res. 2019;19(1):512.	Not PHC reform
253	Zeng XY, Zhang M, Li YC, Huang ZJ, Wang LM. Study on effects of community-based management of hypertension patients aged >/=35 years and influencing factors in urban and rural areas of China, 2010. Zhonghua Liu Xing Bing Xue Za Zhi. 2016;37(5):612-7.	Not PHC reform
254	Zeng Y, Li J, Yuan Z, Fang Y. The effect of China's new cooperative medical scheme on health expenditures among the rural elderly. Int J Equity Health. 2019;18(1):27.	Not PHC reform
255	Zhang H, Hu H, Wu C, Yu H, Dong H. Impact of China's Public Hospital Reform on Healthcare Expenditures and Utilization: A Case Study in ZJ Province. PLoS One. 2015;10(11):e0143130.	Not PHC reform
256	Zhang H, van Doorslaer E, Xu L, Zhang Y, van de Klundert J. Can a results-based bottom-up reform improve health system performance? Evidence from the rural health project in China. Health Econ. 2019;28(10):1204-19.	Not PHC reform
257	Zhang Y, Dong D, Xu L, Miao Z, Mao W, Tang S. Equity in health care after 10 years of the New Rural Co-operative Medical Insurance Scheme in China: an analysis of national survey data. The Lancet. 2018;392.	Not PHC reform
258	Zhou Z, Zhou Z, Gao J, Yang X, Yan J, Xue Q, et al. The effect of urban basic medical insurance on health service utilisation in Shaanxi Province, China: a comparison of two schemes. PLoS One. 2014;9(4):e94909.	Not PHC reform
259	Zhou Z, Zhu L, Zhou Z, Li Z, Gao J, Chen G. The effects of China's urban basic medical insurance schemes on the equity of health service utilisation: evidence from Shaanxi Province. Int J Equity Health. 2014;13:23.	Not PHC reform

260	Chen H, Ning J. The impacts of long-term care insurance on health care utilization and expenditure: evidence from China. Health Policy and Planning. 2022 Jan 15.	Not PHC reform
261	Dong Y, Chen J, Jing X, Shi X, Chen Y, Deng X, et al. Impact of capitation on outpatient expenses among patients with diabetes mellitus in Tianjin, China: a natural experiment. BMJ Open. 2019;9(6):e024807.	Not PHC reform
262	Guo H, Wang X, Xu J, Mao T, Chen J. Prevention of hypertension in patients with prehypertension in the rural areas of China: a community-based quasi-experiment. The Lancet. 2018;392.	Not PHC reform
263	Lei X, Bai C, Hong J, Liu H. Long-term care insurance and the well-being of older adults and their families: Evidence from China. Social Science & Medicine. 2022 Mar 1;296:114745.	Not PHC reform
264	Tang Y, Chen T, Zhao Y, Taghizadeh-Hesary F. The Impact of the Long-Term Care Insurance on the Medical Expenses and Health Status in China. Frontiers in Public Health. 2022:876.	Not PHC reform
265	Xu M, Pei X. Does coinsurance reduction influence informer-sector workers' and farmers' utilization of outpatient care? A quasi-experimental study in China. BMC health services research. 2022 Dec;22(1):1-9.	Not PHC reform
266	叶美荣, 谢兴潜, 周青, 钱昌, 陈蕴辉. 贫困精神病患者社区医疗救助政策调整效果评估. 中国民康医学. 2014;26(6):83-5.	Not PHC reform
267	周强, 张全红, 蔡智全. 农村医疗保险制度对居民收入差距的影响. 中南财经政法大学学报. 2021.	Not PHC reform
268	张艳春, 张丽芳, 秦江梅, 刘涵, 吴宁. 东中西部城市卫生筹资再分配效应比较: 基于社区卫生综合改革典型城市居民健康询问调查. 中国卫生经济. 2013;32(11):51-3.	Not PHC reform
269	李明媚. 社区卫生服务改革对某社区居民卫生资源利用率的影响. 上海预防医学. 2009;21(6):284-6.	Not PHC reform
270	李竞吾, 张伟, 陈田园. 基本医疗保险能显著缓解因病致贫吗——基于不同发展阶段的政策效果比较. 金融经济学研究. 2021.	Not PHC reform
271	杜娟, 徐俊杰, 邵爽, 于海兰, 王慧丽, 赵亚利, et al. 北京市社区卫生服务机构基本药物 "零差率" 政策对农村糖尿病患者治疗费用的影响. 中国医药导报. 2012;9(35):134-6.	Not PHC reform
272	杨贺, 穆怀中. 新农合对农村居民健康水平影响的实证研究. 地方财政研究. 2021.	Not PHC reform

273	王正文, 尹红莉, 崔靖茹. 基本医疗保险制度对农村中老年居民生活质量的影响研究. 中国软科学. 2022.	Not PHC reform
274	许建, 陈丽, 梁小云, 金承刚, 李佳. 社区卫生服务中心收支两条线改革对居民慢性病知识知晓情况的影响研究. 中国全科医学. 2012;15(19):2157-9.	Not PHC reform
275	郑莉, 梁小云. 城乡居民大病保险对灾难性卫生支出的影响. 中国卫生经济. 2021.	Not PHC reform
276	郑超, 王新军, 孙强. 城乡医保统筹政策, 健康风险冲击与精准扶贫绩效研究. 公共管理学报. 2022.	Not PHC reform
277	郑超, 王新军, 孙强. 城乡医保统筹政策, 居民健康及其健康不平等研究. 南开经济研究. 2021(4).	Not PHC reform
278	郑超, 王新军, 孙强. 政府卫生支出的健康绩效研究——基于中国老年健康调查 (CLHLS) 数据的评估. 世界经济文汇. 2022;1(03):103.	Not PHC reform
279	郭庆, 吴忠. 城乡居民医保制度统筹会产生促健防贫效用?——基于 PSM-DID 方法的研究. 中国卫生政策研究. 2020;13(7):7-14.	Not PHC reform
280	鄢洪涛, 杨仕鹏. 农村医疗保险制度的相对贫困治理效应——基于贫困脆弱性视角的实证分析. 湖南农业大学学报 (社会科学版). 2021.	Not PHC reform
281	顾鑫, 周延, 张旭. 城镇居民医疗保险制度的反贫困效果研究. 江西财经大学学报. 2021(2):66.	Not PHC reform
282	马晓, 夏梦, 常玉娥, 于海淼, 张欣, 张柠. 农村社区卫生服务机构收支两条线管理实施效果调查分析. 中国初级卫生保健. 2011;25(12):28-9.	Not PHC reform
283	马桂峰, 盛红旗, 马安宁, 王培承, 安洪庆, 王宪祥, et al. 新型农村合作医疗实施前后乡镇卫生院效率变化的研究. 中国卫生经济. 2012;31(4):52-.	Not PHC reform
284	高健, 丁静. 新农合大病保险能缓解农村长期贫困吗?——来自贫困脆弱性视角的检验. 兰州学刊. 2021.	Not PHC reform
285	周新发, 石安其琛. 医疗保险对减轻参保农村居民家庭就医负担的异质性研究——来自 CFPS 数据的经验证据. 财经理论与实践. 2021;42(05):50-6.	Not PHC reform

286	朱涵涵. 城乡居民基本医疗保险对农村居民医疗服务利用的影响研究——基于 CHARLS 数据的实证分析. 农村经济与科技. 2022;33(09):179-82.	Not PHC reform
287	赵建国, 温馨. 城乡居民基本医疗保险对儿童健康的影响——基于中国家庭追踪调查数据的实证研究. 社会保障研究. 2021.	Not PHC reform
288	邵芯苗, 郭庆, 吴忠. 城乡居民医疗保险对流动人口的健康促进效用研究. 现代预防医学. 2021;48(20):3754-9+63.	Not PHC reform
289	郑超, 王新军, 孙强. 城乡医保统筹政策、健康风险冲击与精准扶贫绩效研究. 公共管理学报. 2022;19(01):146-58+76.	Not PHC reform
290	郭庆, 吴忠. 基于 PSM-DID 模型考察城乡居民基本医疗保险对流动人口的促健防贫效用. 中国卫生统计. 2022;39(02):234-7+42.	Not PHC reform
291	Li YJ, Zou ZH, Yi SY, Yu CY. Effects of community management combined with self-management on treatment compliance and quality of life in elderly patients with type 2 diabetes. Chinese General Practice. 2018 Sep 15;21(26):3217.	Not PHC reform
292	Li Z, Wang Y, Jin C. Effects of Community Health Reform on Prescription of Antibiotics in the Community Health Center of Bao'an District of Shenzhen,through an Interrupted Time Series Design with a Comparison Group. Chinese General Practice. 2018;21(30):3745-8.	Small scale studies
293	Liu Z, Shi L, Kuang H, Yu B, Lei Y. An investigation of the effect of integrated drug management and intervention on drug usage in community patients with chronic diseases. Chinese General Practice. 2018;21(36):4498-501.	Small scale studies
294	Si W, Chen X, Ye R, Liu K, Liu Z, Peng Z, et al. A4597 Effect of three-grade management on the blood pressure control rate in community hypertensive patients under the West China Hospital urban medical service alliance. Journal of Hypertension. 2018;36(Supplement 3):e297.	Small scale studies
295	Su M, Zhou Z, Si Y, Wei X. Effect of health alliances on the quality of primary care in urban China: a coarsened exact matching difference-in-differences analysis. The Lancet. 2019;394(Supplement 1):S86.	Small scale studies
296	Wu HF, Wen CY, Kong XY, Shen L, Wu XY, Wu WW, et al. Effectiveness of Self-Management Patient Education Combined with Family Doctor Team Service among Chinese Type 2 Diabetic Patients in Urban-Rural Fringe Area: A Randomised Controlled Trial. Journal of Hypertension. 2018;36(Supplement 3):E307-E.	Small scale studies
297	Zhao G, Chen S, Hu R. Evaluation on effect of hospital-commmity joint intervention for contraception and birth control in lactation. Chinese Nursing Research. 2015;29(8B):2889-90.	Small scale studies

298	Chen J, Lu J, Wu H, Wu Y, Tan Y, Zhao Z, et al. A Comparative Study of the Effects of Payment Methods by Medical Insurance on Self-management Behavioral Outcomes in Type 2 Diabetic Patients in Guangzhou and Macau. Chinese General Practice. 2019;22(22):2709-12.	Small scale studies
299	刘君, 王凤霞, 张玉娟, 李燕润, 李明霞, 金进, et al. 乡镇卫生院规范化健康教育对提高高血压患者疾病认知度的效果研究. 中国社会医学杂志. 2013;30(4):278-80.	Small scale studies
300	张田, 苗豫东, 王留义, 赵琳, 卢跃峰, 顾建钦. 医联体全科团队对家庭医生签约服务患者体验的影响研究. 中国全科医学. 2019;22(31):3792.	Small scale studies
301	李婷, 林其意, 黄涛, 易春涛. 慢性病长处方政策对签约患者医疗质量的短期影响及政策建议. 中国全科医学. 2017;20(25):3084.	Small scale studies
302	沈艺. 医联体全科团队对居民家庭医生签约意愿的研究. 中国社区医师. 2020.	Small scale studies
303	莫瑞豪, 曾润颜, 黄胜海, 邹敏莉, 亢黎莉. 深圳市福田区家庭医生制服务对居民社区首诊率的影响研究. 中国全科医学. 2015;18(10):1142.	Small scale studies
304	赵文穗, 季黎明, 邱琼, 李黎, 潘向东, 于爱华, et al. 家庭医生制下糖尿病双向转诊效果评估. 中华全科医学. 2015.	Small scale studies
305	赵桂萍, 陈思羽, 胡蕊. 医院-社区联合干预对哺乳期避孕节育的效果评价. 护理研究: 中旬版. 2015;29(8):2889-90.	Small scale studies

Reference

1. He Y, Shen Y, Wang Y, Sang X, Wang P, Ma G. Comparative Analysis on the Operation Status of Township Hospitals before and after the Implementation of Essential Medicine System. *Chinese Health Economics*. 2014;**33**:54-57.
2. Zhu Z, Zhao M, Sun G, Sun H, Yang H. Evaluation of Interventional Effects of Family Doctor Services on Community Residents in Beijing. *Chin Gen Pract*. 2017;**20**:1549-53.
3. Han C, Li C, Li J, Cao Z, Han C. Effects of Essential Drug Zero-profits Sales to Village Clinic Based on the Evaluation Method of Difference-in-Differences. *Chin Gen Pract*. 2016;**19**:2647-50.
4. Ma A, Li C, Shen Y, Shen H, Ma G. Study on the Changes of Hospitalization Costs for Patients in Township Hospitals before and after the Implementation of Essential Medicine System. *Chinese Health Economics*. 2014;**33**:31-34.
5. Wang X, Wang C, Sang X, Wang L, Ma A. Study on the Impact of Essential Medical System on the Medical Services of Township Hospitals. *Chinese Health Economics*. 2014;**33**:42-45.
6. Chen M, Mao W, Chen W, Jiang H, Zhang L. Impact of the essential medicines policies on operation and expenditure burden at primary health care institutions. *Chinese Health Resources*. 2013;**16**:93-94.
7. Li K, Sun Q, Zuo G, Yang H, Meng Q. Study of the Impact of Essential Medicine System on the Patient Visits and Cost in Township Hospitals: Based on the Evaluation Method of Difference in Difference. *Chinese Health Economics*. 2012;**31**:62-64.
8. Duan H, Zhang Y, Hou Y, Ma L. Reseach on the Influence of the Policy of Medical Alliance on the Service Ability of Primary Community Medical and Health Institutions. *Journal of Gansu Administration Institute*. 2020:4-16.
9. Tan H, Chen F, Jing S. Analysis on The Performance Related-pay Impact on Effect of Basic Public Health Services - An Empircial Study Based on the DID. *Chinese Health Service Management*. 2015:68-70.
10. Jin C, Yang H, Liang X, Wu N, Li Z, Chen L. The Impact Evaluation of Essential Medicine Policies on Joint Usage of Antibiotics, Steroids and Intravenous Infusion in Township Health Care. *China Health Economics*. 2013;**32**:53-55.
11. Chen M, Wang L, Chen W, Zhang L, Jiang H, Mao W. Does economic incentive matter for rational use of medicine? China's experience from the essential medicines program. *Pharmacoeconomics*. 2014;**32**:245-55.
12. Ding L, Wu J. The Impact of China's National Essential Medicine Policy and Its Implications for Urban Outpatients: A Multivariate Difference-in-Differences Study. *Value Health*. 2015;**18**:A72-A73.
13. Gong Y, Yang C, Yin X, Zhu M, Yang H, Wang Y, et al. The effect of essential medicines programme on rational use of medicines in China. *Health Policy Plan*. 2016;**31**:21-7.
14. Jiang Q, Jiang Z, Xin Z, Cherry N. Evaluation of the impact of a chronic disease scheme reimbursing medical costs of patients with diabetes in Anhui province, China: a follow-up study. *BMC Public Health*. 2016;**16**:980.
15. Liang X, Xia T, Zhang X, Jin C. Governance structure reform and antibiotics prescription in community health centres in Shenzhen, China. *Fam Pract*. 2014;**31**:311-8.

16. Liu Y, Yuan Z, Liu Y, Jayasinghe UW, Harris MF. Changing community health service delivery in economically less-developed rural areas in China: impact on service use and satisfaction. *BMJ Open*. 2014;**4**:e004148.
17. Shen M, He W, Li L. Incentives to use primary care and their impact on healthcare utilization: Evidence using a public health insurance dataset in China. *Soc Sci Med*. 2020;**255**:112981.
18. Sun J, Zhang X, Zhang Z, Wagner AK, Ross-Degnan D, Hogerzeil HV. Impacts of a new insurance benefit with capitated provider payment on healthcare utilization, expenditure and quality of medication prescribing in China. *Trop Med Int Health*. 2016;**21**:263-74.
19. Tang Y, Liu C, Zhang Z, Zhang X. Effects of prescription restrictive interventions on antibiotic procurement in primary care settings: a controlled interrupted time series study in China. *Cost Eff Resour Alloc*. 2018;**16**:1.
20. Wei X, Li H, Yang N, Wong SY, Chong MC, Shi L, et al. Changes in the perceived quality of primary care in Shanghai and Shenzhen, China: a difference-in-difference analysis. *Bull World Health Organ*. 2015;**93**:407-16.
21. Xu J, Powell-Jackson T, Mills A. Effectiveness of primary care gatekeeping: difference-in-differences evaluation of a pilot scheme in China. *BMJ Glob Health*. 2020;**5**:e002792.
22. Yang L, Huang C, Liu C. Distribution of essential medicines to primary care institutions in Hubei of China: effects of centralized procurement arrangements. *BMC Health Serv Res*. 2017;**17**:727.
23. Yang L, Liu C, Ferrier JA, Zhou W, Zhang X. The impact of the National Essential Medicines Policy on prescribing behaviours in primary care facilities in Hubei province of China. *Health Policy Plan*. 2013;**28**:750-60.
24. Yao Y, Liu GG, Cui Y. Job training and organizational performance: Analyses from medical institutions in China. *China Economic Review*. 2020;**60**.
25. Yin J, Wei X, Li H, Jiang Y, Mao C. Assessing the impact of general practitioner team service on perceived quality of care among patients with non-communicable diseases in China: a natural experimental study. *Int J Qual Health Care*. 2016;**28**:554-60.
26. Zhang D, Pan X, Li S, Liang D, Hou Z, Li Y, et al. Impact of the National Essential Public Health Services Policy on Hypertension Control in China. *Am J Hypertens*. 2017;**31**:115-23.
27. Zhang L, Wang Z, Qian D, Ni J. Effects of changes in health insurance reimbursement level on outpatient service utilization of rural diabetics: evidence from Jiangsu Province, China. *BMC Health Serv Res*. 2014;**14**:185.
28. Zhang X, Wu Q, Liu G, Li Y, Gao L, Guo B, et al. The effect of the National Essential Medicines Policy on health expenditures and service delivery in Chinese township health centres: evidence from a longitudinal study. *BMJ Open*. 2014;**4**:e006471.
29. Zhou Z, Zhao Y, Shen C, Lai S, Nawaz R, Gao J. Evaluating the effect of hierarchical medical system on health seeking behavior: A difference-in-differences analysis in China. *Soc Sci Med*. 2021;**268**:113372.
30. Miao Y, Yuan X, Gu J, Zhang L, He R, Sandeep S, et al. Constructing a value-based healthcare system for hypertensive patients through changing payment mode: evidence from a comparative study in rural China. *J Med Econ*. 2019;**22**:245-51.

31. Powell-Jackson T, Yip WC, Han W. Realigning demand and supply side incentives to improve primary health care seeking in rural China. *Health Econ*. 2015;**24**:755-72.
32. Shen M, He W, Yeoh EK, Wu Y. The association between an increased reimbursement cap for chronic disease coverage and healthcare utilization in China: an interrupted time series study. *Health Policy Plan*. 2020;**35**:1029-38.
33. Yi H, Miller G, Zhang L, Li S, Rozelle S. Intended and unintended consequences of China's zero markup drug policy. *Health Aff (Millwood)*. 2015;**34**:1391-8.
34. Miao Y, Gu J, Zhang L, He R, Sandeep S, Wu J. Improving the performance of social health insurance system through increasing outpatient expenditure reimbursement ratio: a quasi-experimental evaluation study from rural China. *Int J Equity Health*. 2018;**17**:89.
35. Sun X, Liu X, Sun Q, Yip W, Wagstaff A, Meng Q. The Impact of a Pay-for-Performance Scheme on Prescription Quality in Rural China. *Health Econ*. 2016;**25**:706-22.
36. Miao Y, Zhang L, Sparring V, Sandeep S, Tang W, Sun X, et al. Improving health related quality of life among rural hypertensive patients through the integrative strategy of health services delivery: a quasi-experimental trial from Chongqing, China. *Int J Equity Health*. 2016;**15**:132.
37. Liu C, Zhang X, Wang X, Zhang X, Wan J, Zhong F. Does public reporting influence antibiotic and injection prescribing to all patients? A cluster-randomized matched-pair trial in china. *Medicine (Baltimore)*. 2016;**95**:e3965.
38. Hu H, Liang H, Wang H. Longitudinal study of the earliest pilot of tiered healthcare system reforms in China: Will the new type of chronic disease management be effective? *Soc Sci Med*. 2021;**285**:114284.
39. Wang L, Liu W. Effects of Family Doctor Contract Services on the Health-Related Quality of Life Among Individuals With Diabetes in China: Evidence From the CHARLS. *Front Public Health*. 2022;**10**:865653.
40. Yuan S, Fan F, Zhu D. Effects of Vertical Integration Reform on Primary Healthcare Institutions in China: Evidence From a Longitudinal Study. *Int J Health Policy Manag*. 2021;**11**:1-9.
41. Shen C, Zhou Z, Lai S, Dong W, Zhao Y, Cao D, et al. Whether high government subsidies reduce the healthcare provision of township healthcare centers in rural China. *BMC Health Serv Res*. 2021;**21**:1184.
42. Pan C, Yang J. The Impact of the Implementation of Hierarchical Medical Policy on Health Inequality among the Chinese Elderly. *Social Security Studies*. 2022:49-60.
